# Contribution to the knowledge of the bee fauna (Hymenoptera, Apoidea, Anthophila) in Serbia

**DOI:** 10.3897/zookeys.1053.67288

**Published:** 2021-08-02

**Authors:** Sonja Mudri-Stojnić, Andrijana Andrić, Zlata Markov-Ristić, Aleksandar Đukić, Ante Vujić

**Affiliations:** 1 University of Novi Sad, Faculty of Sciences, Department of Biology and Ecology, Trg Dositeja Obradovića 2, 21000 Novi Sad, Serbia; 2 University of Novi Sad, BioSense Institute, Dr Zorana Đinđića 1, 21000 Novi Sad, Serbia; 3 Scientific Research Society of Biology and Ecology Students “Josif Pančić”, Trg Dositeja Obradovića 2, 21000 Novi Sad, Serbia

**Keywords:** diversity, fauna, Serbia, wild bees

## Abstract

The current work represents summarised data on the bee fauna in Serbia from previous publications, collections, and field data in the period from 1890 to 2020. A total of 706 species from all six of the globally widespread bee families is recorded; of the total number of recorded species, 314 have been confirmed by determination, while 392 species are from published data. Fourteen species, collected in the last three years, are the first published records of these taxa from Serbia: *Andrenabarbareae* (Panzer, 1805), *A.clarkella* (Kirby, 1802), *A.fulvicornis* (Schenck, 1853), *A.intermedia* (Thomson, 1870), *A.lapponica* (Zetterstedt, 1838), *A.pandellei* (Pérez, 1895), *A.paucisquama* (Noskiewicz, 1924), *A.simillima* (Smith, 1851), *Panurginusherzi* (Morawitz, 1892), *Epeoloidescoecutiens* (Fabricius, 1775), *Nomadaleucophthalma* (Kirby, 1802), *Chelostomanasutum* (Pérez, 1895), *Hoplitisclaviventris* (Thomson, 1872), and *Dasypodapyrotrichia* (Förster, 1855). Almost all the species recorded so far in Serbia belong to the West-Palaearctic biogeographical region, except *Megachilesculpturalis* (Smith, 1853), which is an alien invasive species native to East Asia. According to the European Red List of bees, 221 species listed in this paper were assessed as Data Deficient; threatened species mostly belong to the families Apidae with 13 species, Colletidae with eight species, and Halictidae with five species. This study contributes to the knowledge of the distribution of bee species in Europe. The present work provides a baseline for future research of wild bee diversity in Serbia and neighbouring regions at the local and regional levels, and a basis for their conservation.

## Introduction

The first available data on the faunistic research of Hymenoptera: Apoidea: Anthophila in the Balkan countries derives from the late 19^th^ century, from the period of the Austro-Hungarian Empire. At that time, scientists were collecting data on the wild bee fauna while travelling through the area of today’s Balkan countries, or they researched parts of the countries where they lived. The borders and names of the Balkan countries have changed several times since then. Therefore, in the present paper, the localities are shown within the current borders of the Republic of Serbia.

The earliest publication, which provided the data on the bee fauna of Serbia is by Korlević (1890), who recorded 15 bee species in the Pannonian Region of the country. Six years later, Apfelbeck (1896) collected specimens of 101 wild bee species in southeast Serbia during his travels through Balkan countries. Soon after that, Mocsáry (1897) published “Fauna Regni Hungarie”. This publication contains significant data from Deliblato Sands in today’s Serbia, where 199 wild bee species were recorded. Vorgin (1918) provided data on the bee fauna from Fruška Gora Mountain and several sites on the margins of the Danube, where 97 bee species were recorded, and for the first time from all families of Apoidea. The most reliable and comprehensive publication on the bee fauna of Serbia was published by Lebedev (1931). Bee specimens were collected across several Balkan countries by more than ten experts and encompassed 258 bee species from former Yugoslavia, among which 203 were from Serbia. Most of these bees belong to Central European species and the whole fauna was very similar to that of Hungary and Romania (Lebedev 1931). Vorgin (1955) published contributions to the fauna Hymenoptera: Apoidea: Anthophila of Yugoslavia, including new data on bee fauna and sites for 114 bee species, which were recorded in Serbia. Rafajlović and Seleši (1958) presented data from Alfred Taubert’s collection, who collected insects for 35 years (1909–1944) in the territory of former Yugoslavia. In Serbia, 267 bee species were recorded mostly in the Vojvodina province, in the vicinity of the city of Subotica and Deliblato Sands. The value of the collection lies in the fact that Taubert collected twice as many species as Mocsáry (1897) in the same area (Rafajlović and Seleši 1958).

After World War II, many authors mainly focused their research on studying the ecology or life history and biology of some species, or the bee diversity of certain regions. Grozdanić studied the life history and ecology of *Lasioglossummarginatum* (Brullé, 1832) (as *Halictusmarginatus* Brullé, 1832), *L.interruptum* (Panzer, 1798) (as *H.interruptus* (Panzer, 1798)), *L.malachurum* (Kirby, 1802) (as *H.malachurus* (Kirby, 1802)), *H.sajoi* (Blüthgen, 1923), *H.maculatus* (Smith, 1848), *H.asperulus* (Pérez, 1895), *Seladoniakessleri* (Bramson, 1879) (as *H.kessleri* Bramson, 1879), *Osmiabicornis* Linnaeus, 1758, *O.bicolor* (Schrank, 1781), *O.rufohirta* (Latreille, 1811), *O.bidentata* (Morawitz, 1876), *Xylocopaviolacea* (Linnaeus, 1758), *X.valga* (Gerstäcker, 1872), *X.iris* Christ, 1791 (as *X.cyanescens* Brullé, 1832), *Bombuspascuorum* Scopoli, 1763 (as *B.agrorum* (Fabricius, 1787)), *Apismellifera* (Linnaeus, 1758), *Anthophoraplumipes* Pallas, 1772 (as *A.acervorum* (Linnaeus, 1758)), *A.plagiata* Illiger, 1806 (as *A.parietina* (Fabricius, 1793)), *A.salviae* (Panzer, 1804) (as *A.crinipes* Smith, 1854), *A.pubescens* (Fabricius, 1781), Eucera (Tetralonia) lyncea (as *Tetralonialyncea* (Mocsáry, 1879)), Eucera (Tetralonia) nana (as *T.nana* (Morawitz, 1874)), *Systrophaplanidens* (Giraud, 1861), *S.curvicornis* (Scopoli, 1770), *Euceraexcisa* (Mocsáry, 1879), *Megachileericetorum* (Lepeletier, 1841), and *Ceratina* spp. (Grozdanić 1926, 1928, 1930, 1950a, b, 1956, 1958a, b, 1960, 1961, 1965, 1966, 1968, 1969a–c, 1970, 1971a, b, 1972a, b, 1974; Grozdanić and Čolović 1955a, b; Grozdanić and Stevanović 1959, 1965; Grozdanić and Krunić 1961; Grozdanić and Vasić 1965a, b, 1966a, b, 1967a, b, 1968, 1970; Grozdanić and Mučalica 1966, 1968a, b, 1969, 1973; Grozdanić and Baranov 1963; Grozdanić and Radivojevic 1972). Grozdanić and Vasić (1965c, 1966a) published two papers on their entomological research in the vicinity of Belgrade and their faunistic lists contained 35 bee species. Živojinović (1950) provided a comprehensive monograph on the fauna of insects from eastern Serbia and recorded 112 bee species in the Majdanpek region, on the southern Carpathian Mountain. Petrik (1958) published the results of a two-year study of the insect fauna in the area of Deliblato Sands, where he recorded 58 bee species. Vasić studied the life history and biology of *Lasioglossummarginatum* (as *Halictusmarginatus*), *H.quadricinctus* (Fabricius, 1776), *H.scabiosae* (Rossi, 1790), *Megachilealbisecta* Klug, 1817 (as *Megachilesericans* Fonscolombe, 1832) (Vasić 1966, 1967, 1968, 1970, 1979a, b). Mučalica, Z. studied the biology and life history of *Halictusfulvipes* (Klug, 1817), *Anthophoraplagiata* (as *A.parietina*), and *A.salviae* (as *A.crinipes*) (Mučalica 1968, 1984, 1987a, b, 1990, 1997). Mučalica and Stanivljević (2005) studied the nesting biology of *Megachilewillughbiella* (Kirby, 1802). Grozdanić and Mučalica researched the fauna of Hymenoptera across former Yugoslavia; Mučalica collected insects for 33 years (1965–1997) in the area of Serbia and Grozdanić collected insects in the period from 1963 to 1972. Both collections are preserved in the Natural History Museum in Belgrade. The present paper lists 181 bee species recorded in Serbia by the above authors. Krunić studied two important wild bee pollinators of Serbian orchards, namely *Osmiacornuta* (Latreille, 1805) and *O.bicornis* (as *O.rufa* (Linnaeus, 1758)) in all aspects of their life history such as diapause, overwintering, distribution, population management, etc. (Krunić et al. 1989, 1991, 1992a, 1995a, 1996, 1997, 1998, 1999, 2001, 2005; Krunić and Stanisavljević 2006a–d). The other pollinator bee species he studied was *Megachilerotundata* (Fabricius, 1793) (Krunić et al. 1985, 1992b, c, 1995b, 1997). Krunić, Radović and Brajković (1988) published a list of the Megachilide family collected in former Yugoslavia. Krunić also studied the population of honey bees, mostly in the Pannonian region (Krunić 1967, 1986, 1994; Krunić et al. 1994). Stanisavljević continued research into bee pollinators in orchards, mostly *Osmiacornuta* and *O.bicornis* (as *O.rufa*), from the environmental, conservation, morphology, and management aspects (Stanisavljević 1996, 2000, 2009; Stanisavljević et al. 1997a, b, 1999, 2000a, b, 2013). Stanisavljević and Nedić (2008) published a paper on the role of bees in orchard pollination in Serbia. Stanisavljević (2013) published a list of bee species from the Megachilidae family of Fruška Gora. Stanisavljević and Tomović (2006) presented the results of alfalfa seed production with the use of *Megachilerotundata* in Serbian agricultural farms. Mudri-Stojnić studied bee fauna in agro-ecosystems of Vojvodina province from 2011. Markov studied bee fauna in protected areas of Vojvodina province (Markov et al. 2016) and their economic aspect (Markov 2017). Đukić studied the bee fauna of the Vlasina region in southeast Serbia; in two years (2019–2020), he recorded 99 bee species.

The importance of bees in terrestrial ecosystems, as well as their ecosystem role in the process of pollination of agricultural crops and wild plants, is widely known. In many European countries, Red Data Books or Red Lists of bees have been produced at the national level. Some European countries have developed specific national actions in order to enhance bee populations and to arrest decline, introduced legislation with the aim of legally protecting all or some species of bees, and/or produced checklists of bees. For some Balkan countries, such as Serbia, data on the diversity of bees are scarce. Among the reasons for such a situation are an insufficient number of wild bee experts and the absence of proper collections. Up-to-date entomological research programmes of wild bees have not been spatially systematic, so certain areas of Serbia have been studied more, while others less. Although there is clear evidence of a decline in pollinators diversity and abundance across Europe (Potts et al. 2010; Nieto et al. 2014; Goulson et al. 2015), there are no initiatives or activities to protect wild bee species or their habitats in Serbia.

The present study summarises for the first time all the available records of species of wild bees in Serbia. This paper is not intended as a national checklist of bees, since there are undoubtedly more species yet to be found. The aims of this study are: 1) to review the records on the bee fauna, according to bibliographic sources known to the authors, 2) to present some more recent observations, and finally 3) to provide an updated preliminary list of the species of bees occurring in Serbia. The major purpose of this article is to broaden the knowledge of bee diversity in Serbia and pave the way for future research of wild bee fauna at local and regional levels. Another important aim is to improve an understanding of the status and trends of European pollinators.

## Materials and methods

### Study area

Serbia is situated in central and southeast Europe, mostly in the central Balkan peninsula, while its northern part spreads over the southern belt of the Pannonian Plain. The country’s total area is 88,361 km² (Spatial Plan RS 2021–2035, Official Gazette of RS No. 48/19). The main geographic units in Serbia are the Pannonian Region in the north, which covers a third of the country, the Peripannonian Region in the central part of the country, which chiefly consists of hills traversed by rivers, and the mountain and basin region which are dominant in the south. The Carpathian Mountains and the Balkan Mountains stretch in the north-south direction through east Serbia. The Dinaric Arc stretches in the west and southwest. The climate of Serbia is under the influences of the landmass of Eurasia, the Atlantic Ocean, and the Mediterranean Sea. It classifies as a warm-humid continental or humid subtropical climate. In the north of the country, the climate is more continental, whereas south and southeast Serbia are influenced by the Mediterranean climate (Stevanović and Stevanović 1995). In the north of Serbia, the Pannonian Plain is a lowland landscape with large rivers (e.g. the Danube, Sava, and Tisa) while to the south hilly or mountainous landscapes are intersected by river valleys. In Vojvodina, there is a large sandy area called Deliblato Sands, which is rare and unusual in inland Europe. The main habitat types are: steppe grasslands and wooded steppe, mesophilic meadows, saline grassland, shrubs, wetland, mainly deciduous southern European forests, coniferous forests, and high-mountain rocky areas and pastures.

Serbia is a country with a number of rich ecosystems, and species diversity of many groups of organisms is high and contributes to a significant part of Europe’s biodiversity. According to previous research, in the territory of the Republic of Serbia there are: 39% of the European vascular flora, 74% of the European bird fauna, 67% of the European mammal fauna, 51% of the European fish fauna, and 49% of the European reptile and amphibian fauna (IUCN 2021). Conserved habitats, from lowland grasslands and wetlands, through forests and other higher habitats, to high mountain areas intersected with gorges and major lowland rivers (e.g. the Danube, Sava, and Tisa), all form the basis for its biodiversity. There are 462 protected areas in Serbia on 7.65% of its surface, among which are five National Parks, 18 Nature Parks, 20 Protected Landscapes, 68 Nature Reserves, six Protected Habitats, and 308 Natural Monuments (Spatial Plan RS 2021–2035, Official Gazette of RS No. 48/19). The ecological network that consists of ecologically significant areas and ecological corridors covers 101 nationally and internationally significant areas, comprising 21% of the total area of Serbia. Most areas within the ecological network have an international status based on several aspects: 61 Emerald Areas of Special Conservation Importance – ASCI; 42 Important Bird Areas – IBA; 61 Important Plant Areas – IPA; 40 Prime Butterfly Areas – PBA; ten Ramsar sites. The ecological network also includes other spaces and places that have yet to be spatially identified (Spatial Plan RS 2021–2035, Official Gazette of RS No. 48/19). Agricultural production on annual crops is mostly present in the Pannonian Plain. Serbia produces various agricultural products, mostly grains, fruits, and vegetables. According to the FAOSTAT, Serbia is among the top five world producers of raspberries and plums, which are mainly produced in the southwest. Agricultural landscapes occupy 63.7% of the territory (Spatial Plan RS 2021–2035, Official Gazette of RS No. 48/19).

### Methodology

This paper represents a list of bee species in Serbia based on the compiled data known to the authors, gathered from available entomological collections and literature sources between 1890 and 2020, and our own faunistic studies in the decade 2010–2020. Therefore, it includes previously published and unpublished data, supplied by different specialists, as well as some recent records from the authors of this paper. In total, more than 100 publications were examined for relevant records. Additionally, the present list was based on reviewing a database from the online Checklist of Western Palaearctic Bees (Hymenoptera: Apoidea: Anthophila) by Kuhlmann et al. (2020), which provides basic information on bee diversity in the Serbian region.

The following abbreviations are used in the text:

**AĐ coll.** Aleksandar Đukić private collection (determined by Zsolt Józan, specialist in Aculeata research in Central Europe; bee specialist Dr Andrej Gogala from the Slovenian Museum of Natural History, Ljubljana, Slovenia; Prof. Denis Michez, Laboratory of Zoology, University of Mons, and PhD student Jelle Devalez, Department of Geography, University of the Aegean);

**AZ coll.** Aleksandra Zatezalo collection of the Institute for Nature Conservation of Serbia, Belgrade, Serbia (determined by Zsolt Józan);

**FSUNS**Department of Biology and Ecology, Faculty of Sciences, University of Novi Sad, Novi Sad, Serbia;

**SG coll.** Simeun Grozdanić collection of the NHMB (Natural History Museum, Belgrade), Serbia;

**ZM coll.** Zoran Mučalica collection of the NHMB, Serbia.

The cited PhD thesis of Stanisavljević (2000) is based on the material collected by the author, data from the available private collections and collections of the NHMB, as well as data from the published literature, which refer to the researched area. PhD theses of Markov (2017) and Mudri-Stojnić (2018) are based on the material from the collection of the FSUNS. The material from the FSUNS entomological collection were determined by Zsolt Józan. The cited Živojinović (1950) and Petrik (1958) collections are not preserved. The paper by Vorgin (1955) was based on data from the collection of the Croatian Natural History Museum, whose data had been collected for ca. 50 years. The data on registered species from the genera *Andrena* (Fabricius, 1775) and *Bombus* (Latreille, 1802) were not published in the paper by Rafajlović and Seleši (1958); the authors had no knowledge of that collection, which at the time was kept in the Zoological Museum of Zagreb; these data were published by Vorgin (1955). The Alfréd Taubert collection was identified by Alfréd Taubert himself with the help of Paul Blüthgen; unfortunately, professional curation of the collection was not provided, and as a consequence, the collection has not been preserved. Lebedev (1931) compiled data from the collection of the Entomological Institute of Belgrade. The collection contained specimens mostly collected by J. Vagnera and A. Matisena from all over Serbia. The species from the material were determined by Lebedev, P. Blüthgen (*Halictus* sensu lato species), and V. Popov (*Bombus* species). This collection was destroyed during World War II. The publication by Vorgin (1918) contains data from the A. Hensch collection and new data collected by Vorgin, as well as bee collections from the Croatian Natural History Museum. The species from the Apfelbeck, V. (1896) material were determined by H. Friese. The species from the Korlević (1890) material were determined by: A. Mocsary, A. Braunis, F. Koh, H. Friese, F.W. Konow, L. Biró, and G. Mayr. The Anton Korlević entomological collection is housed in the Croatian Natural History Museum.

**Figure 1.**
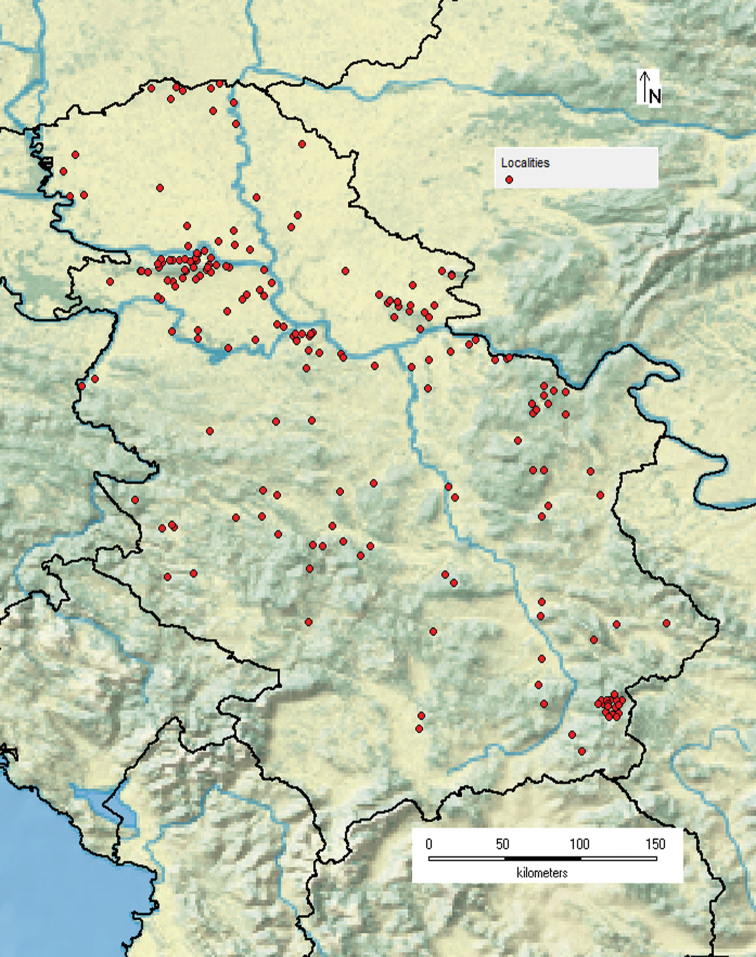
Map of Serbia showing the locations where specimens were collected.

A map of Serbia (Fig. 1) shows the 193 sites where sampling was carried out. The localities were gathered from publications cited in this paper and from data labels in the collections from the Natural History Museum, Belgrade, from the Institute for Nature Conservation of Serbia, and the AĐ collection.

### List format

Nieto et al. (2014) were consulted for the nomenclature of the accepted species names, and the nomenclatural and classification changes suggested by Rasmont et al. (2017) and Dorchin et al. (2018) were adopted. Kuhlmann et al. (2020) and Michez et al. (2019) were consulted for classification and the authorities. For clarification of synonyms and other names mentioned in various cited publications, mostly Kuhlmann et al. (2020), but also other sources, were consulted (NBN 2020; Rasmont and Haubruge 2020; Zicha 2020). The valid species’ names are shown in bold; families, genera, and species are arranged in alphabetical order. For each species, all references providing records are listed by year; if different from the valid name, the name by which the species is referred to in the original publication is written after “as”. Species with no records in Serbia after the 19^th^ century are marked with an asterisk (*). Among the species confirmed by determination of studied material, examined specimens are marked with the double oblique hyphen (⸗) and non-marked species represent records based only on literature data. The black small squares (▪) mark species for which the only source of occurrence in Serbia is the Checklist of the Western Palaearctic Bees (Kuhlmann et al. 2020). At the end of each species paragraph, the IUCN Red List Category (Europe), according to Nieto et al. (2014), is given in square brackets (abbreviations: CR – Critically Endangered, EN – Endangered, VU – Vulnerable, NT – Near Threatened, LC – Least Concern, DD – Data Deficient). Data about the new material examined are given for the specimens that represent the first published records of species for Serbia.

## Results

The list of bees in Serbia presented consists of six families, 58 genera, and 706 species, recorded during the past 130 years. Of the total number of the recorded species, 314 have been confirmed by determination, while 392 species are from literature data. The reported families with the numbers of species (confirmed by determination/based only on literature data) are: Apidae 226 species (91/135), Megachilidae 148 species (62/86), Halictidae 138 species (73/65), Andrenidae 112 species (68/44), Colletidae 69 species (14/55), and Mellitidae 13 species (6/7). The present list includes 14 species with no previously published records for Serbia: *Andrenabarbareae* (Panzer, 1805), *A.clarkella* (Kirby, 1802), *A.fulvicornis* (Schenck, 1853), *A.intermedia* (Thomson, 1870), *A.lapponica* (Zetterstedt, 1838), *A.pandellei* (Pérez, 1895), *A.paucisquama* (Noskiewicz, 1924), *A.simillima* (Smith, 1851), *Panurginusherzi* (Morawitz, 1892), *Epeoloidescoecutiens* (Fabricius, 1775), *Nomadaleucophthalma* (Kirby, 1802), *Chelostomanasutum* (Pérez, 1895), *Hoplitisclaviventris* (Thomson, 1872), and *Dasypodapyrotrichia* (Förster, 1855).

The diversity and proportional representation of bee families are given in Table 1, and the summary of numbers and proportions of bee species within each category of threat according to the European Red List are presented in Table 2.

**Table 1.** Diversity and proportional representation of bee families in Serbia, Europe, and the West Palaearctic region (WP).

Family	Serbia				Europe (Nieto et al. 2014 + Rasmont et al. 2017)		WP* (Rasmont et al. 2017)	
	No of genera	% of 58 genera	No of species	% of 706 species	No of species	% of 1,965+86 species	No of species	% of 3,408 species
Andrenidae	6	10.3	112	15.9	465+24	23.8	716	21.0
Apidae	18	31.0	226	32.0	561+16	28.1	926	27.2
Colletidae	2	3.4	69	9.8	146	7.1	270	7.9
Halictidae	12	20.7	138	19.5	314+30	16.8	585	17.2
Megachilidae	17	29.3	148	21.0	442+14	22.2	852	25.0
Mellitidae	3	5.2	13	1.8	37+2	1.9	59	1.7

*area between 26° and 72° latitude north and from 32° longitude west to 62° longitude east.

**Table 2.** Summary of numbers and proportion of bee species within each category of threat.

The European Red List	Europe (Nieto et al. 2014)		Serbia	
Category	No of species	% of 1,942 species*	No of species	% of 704 species**
Critically Endangered	7	0.4	1	0.1
Endangered	46	2.4	18	2.6
Vulnerable	24	1.2	10	1.4
Near Threatened	101	5.2	64	9.1
Least Concern	663	34.1	390	55.4
Data Deficient	1,101	56.7	221	31.4

*does not include the Not Applicable species in Europe, e.g., species of marginal occurrence (Nieto et al. 2014). **does not include two species not listed in the European Red List.

### Andrenidae (6 genera; 112 species)

*Andrena* Fabricius, 1775 (104 species)

1. ▪ Andrena aberrans Eversmann, 1852 in Kuhlmann et al. (2020). [NT]
2. ⸗ Andrena aeneiventris Morawitz, 1872 in Kuhlmann et al. (2020); Mudri-Stojnić (2018); Markov (2017); Markov et al. (2016); Vorgin (1955); Mocsáry (1897). [LC]
* Andrena albopunctata (Rossi, 1792) in Mocsáry (1897); as Andrena funebris (Panzer, 1798) in Korlević (1890). [LC]
4. ⸗ Andrena alfkenella Perkins, 1914 in Mudri-Stojnić (2018); Lebedev (1931). [DD]
5. Andrena argentata Smith, 1844 in Vorgin (1955); Mocsáry (1897). [DD]
6. Andrena atrata Friese, 1887 in Vorgin (1918); Mocsáry (1897); as Andrena bicarinata (Morawitz, 1876) in Vorgin (1955); Lebedev (1931). [DD]
7. ⸗ Andrena barbareae Panzer, 1805 New material examined: 1 ♀; Vlasina, Veliki Čemernik; 42.7368°N, 22.2723°E; 25 May 2019; M. Vujić leg.; Andrej Gogala det.; AĐ coll. 1 ♀; Vlasina, Vrtop; 42.7904°N, 22.372°E; 20 Jul. 2019; A. Đukić leg.; Andrej Gogala det.; AĐ coll. [DD]
8. Andrena barbilabris (Kirby, 1802) as Andrena sericea Smith, 1791 in Vorgin (1955); as Andrena albicrus (Kirby, 1802) in Mocsáry (1897). [DD]
9. ⸗ Andrena bicolor Fabricius, 1775 in Kuhlmann et al. (2020); as Andrena gwynana (Kirby, 1802) in Živojinović (1950); Lebedev (1931); AĐ coll [LC]
10. Andrena bimaculata (Kirby, 1802) in Kuhlmann et al. (2020); Živojinović (1950); Lebedev (1931). [DD]
11. Andrena brumanensis Friese, 1899 in Kuhlmann et al. (2020); as Andrena clypeata Brullé, 1832 in Živojinović (1950). [LC]
12. Andrena bucephala Stephens, 1846 in Kuhlmann et al. (2020); Živojinović (1950); Lebedev (1931). [DD]
13. ⸗ Andrena carantonica Pérez, 1902 in Kuhlmann et al. (2020); Markov (2017); Markov et al. (2016); ZM coll.; as Andrena jacobi Perkins, 1921 in Lebedev (1931). [DD]
14. ⸗ Andrena chrysopyga Schenck, 1853 in Kuhlmann et al. (2020); Vorgin (1955, 1918); Lebedev (1931); ZM coll. [DD]
15. ▪ Andrena cineraria (Linnaeus, 1758) in Kuhlmann et al. (2020). [LC]
16. ⸗ Andrena clarkella (Kirby, 1802) New material examined: 1 ♀; Vlasina Rid; 42.7253°N, 22.3284°E; 21 Jul. 2019; A. Đukić leg.; Andrej Gogala det.; AĐ coll. [DD]
* Andrena coitana (Kirby, 1802) as Andrena shawella (Kirby, 1802) in Mocsáry (1897). [DD]
18. Andrena colletiformis Morawitz, 1874 in Kuhlmann et al. (2020); Apfelbeck (1896). [DD]
19. ⸗ Andrena combaella Warncke, 1966 in Mudri-Stojnić (2018). [DD]
20. ⸗ Andrena combinata (Christ, 1791) in Kuhlmann et al. (2020); Lebedev (1931); Mocsáry (1897); Apfelbeck (1896); ZM coll. [DD]
21. ▪ Andrena comta Eversmann, 1852 in Kuhlmann et al. (2020). [EN]
22. ⸗ Andrena confinis Stöckhert, 1930 in Markov (2017); Markov et al. (2016); Živojinović (1950).
23. ⸗ Andrena congruens Schmiedeknecht, 1884 in Kuhlmann et al. (2020); AĐ coll. [LC]
24. ⸗ Andrena cordialis Morawitz, 1877 in Kuhlmann et al. (2020); Markov (2017); Markov et al. (2016). [DD]
25. ⸗ Andrena curvana Warncke, 1965 in Markov (2017); Markov et al. (2016). [DD]
26. ▪ Andrena decipiens Schenck, 1861 in Kuhlmann et al. (2020). [DD]
27. Andrena denticulata (Kirby, 1802) in Lebedev (1931). [DD]
28. ⸗ Andrena dorsalis Brullé, 1832 in Kuhlmann et al. (2020); ZM coll. [DD]
29. Andrena dorsata (Kirby, 1802) in Kuhlmann et al. (2020); Živojinović (1950); Lebedev (1931); as Andrena dubitata Schenck, 1870 in Vorgin (1955); Apfelbeck (1896). [DD]
30. Andrena erythrocnemis Morawitz, 1870 in Lebedev (1931); Mocsáry (1897). [DD]
* Andrena fimbriata Brullé, 1832 in Mocsáry (1897). [DD]
32. ⸗ Andrena flavipes Panzer, 1799 in Kuhlmann et al. (2020); Mudri-Stojnić (2018); Markov (2017); Markov et al. (2016); Mudri-Stojnić et al. (2012); Grozdanić (1971b); Vorgin (1955); Živojinović (1950); Lebedev (1931); AZ coll.; ZM coll.; also as Andrena extricata Smith, 1849 in Apfelbeck (1896); as Andrena extricata in Mocsáry (1897); Korlević (1890); as Andrena fulvicrus Dufour, 1841 in Petrik (1958). [LC]
33. ⸗ Andrena florea Fabricius, 1793 in Kuhlmann et al. (2020); Markov (2017); Markov et al. (2016); Apfelbeck (1896); as Andrena rosae var. austriaca Schmied. in Živojinović (1950); as Andrena austriaca Panzer, 1798 in Mocsáry (1897). [DD]
34. ⸗ Andrena fulvago (Christ, 1791) in Kuhlmann et al. (2020); Mudri-Stojnić (2018); Markov (2017); Markov et al. (2016); Mudri-Stojnić et al. (2012); Vorgin (1955); Lebedev (1931). [DD]
35. ⸗ Andrena fulvicornis Schenck, 1853 New material examined: 1 ♀; Beočin, Fruška gora, Časorske livade; 45.1894°N, 19.7451°E; 15 Jun. 2018; S. Mudri-Stojnić leg.; Zsolt Józan det.; FSUNS. [DD]
* Andrena fuscosa Erichson, 1835 as Andrena ephippium Spinola, 1838 in Mocsáry (1897). [DD]
37. ⸗ Andrena gelriae van der Vecht, 1927 in Markov (2017); Markov et al. (2016); AZ coll. [DD]
38. ⸗ Andrena gravida Imhoff, 1832 in Kuhlmann et al. (2020); Lebedev (1931); AĐ coll.; AZ coll.; ZM coll. [DD]
39. Andrena grozdanici Osytshnjuk, 1975 in Kuhlmann et al. (2020); Mučalica (1984). [DD]
40. ⸗ Andrena haemorrhoa (Fabricius, 1781) in Kuhlmann et al. (2020); AĐ coll.; AZ coll.; as Andrena albicans auct. nec Müller in Živojinović (1950); Lebedev (1931). [LC]
41. ⸗ Andrena hattorfiana (Fabricius, 1775) in Kuhlmann et al. (2020); Mudri-Stojnić (2018); Markov (2017); Markov et al. (2016); Mudri-Stojnić et al. (2012); Lebedev (1931); Vorgin (1918); Mocsáry (1897); Apfelbeck (1896); AĐ coll. [NT]
42. Andrena hedikae Jaeger, 1934 in Kuhlmann et al. (2020); Vorgin (1955). [DD]
43. ⸗ Andrena humilis Imhoff, 1832 in Kuhlmann et al. (2020); Lebedev (1931); in Apfelbeck (1896); AĐ coll.; as Andrena fulvescens Smith, 1847 in Vorgin (1955, 1918). [DD]
44. ▪ Andrena hungarica Friese, 1887 in Kuhlmann et al. (2020). [DD]
45. Andrena hypopolia Schmiedeknecht, 1884 in Kuhlmann et al. (2020); Vorgin (1955, 1918); Mocsáry (1897); Korlević (1890). [DD]
46. ⸗ Andrena impunctata Pérez, 1895 in Kuhlmann et al. (2020); Markov (2017); Markov et al. (2016). [LC]
47. ⸗ Andrena intermedia Thomson, 1870 New material examined: 1 ♀; Vlasina, Veliki Čemernik; 42.7368°N, 22.2723°E; 21 Jul. 2019; M. Vujić leg.; Andrej Gogala det.; AĐ coll. [LC]
48. ⸗ Andrena labialis (Kirby, 1802) in Kuhlmann et al. (2020); Mudri-Stojnić (2018); Markov (2017); Markov et al. (2016); Grozdanić (1970); Vorgin (1955, 1918); Živojinović (1950); Lebedev (1931); Mocsáry (1897); Apfelbeck (1896); AZ coll.; ZM coll. [DD]
49. ⸗ Andrena labiata Fabricius, 1781 in Lebedev (1931); ZM coll. [DD]
50. ▪ Andrena lagopus Latreille, 1809 in Kuhlmann et al. (2020). [LC]
51. ⸗ Andrena lapponica Zetterstedt, 1838 New material examined: 1 ♀; Vlasina, Gadžini; 42.7378°N, 22.3042°E; 25 May 2019; A. Đukić leg.; Andrej Gogala det.; AĐ coll. [LC]
52. ⸗ Andrena lathyri Alfken, 1899 in Kuhlmann et al. (2020); Grozdanić (1970); ZM coll. [DD]
53. ⸗ Andrena limata Smith, 1853 in Mudri-Stojnić (2018); as Andrena pectoralis Schmiedeknecht, 1883 in Vorgin (1955); Mocsáry (1897); Korlević (1890). [DD]
54. Andrena limbata Eversmann, 1852 in Lebedev (1931). [DD]
55. Andrena marginata Fabricius, 1776 in Kuhlmann et al. (2020); Lebedev (1931); Mocsáry (1897). [DD]
56. ▪ Andrena mehelyi Alfken, 1936 in Kuhlmann et al. (2020). [DD]
57. ⸗ Andrena minutula (Kirby, 1802) in Kuhlmann et al. (2020); Markov (2017); Markov et al. (2016); Lebedev (1931); AĐ coll.; as Andrena parvula (Kirby, 1802) in Apfelbeck (1896). [DD]
58. ⸗ Andrena minutuloides Perkins, 1914 in Kuhlmann et al. (2020); Mudri-Stojnić (2018); Markov (2017); Markov et al. (2016); Lebedev (1931). [DD]
59. ⸗ Andrena mocsaryi Schmiedeknecht, 1884 in Markov (2017); Markov et al. (2016). [LC]
60. Andrena morio Brullé, 1832 in Kuhlmann et al. (2020); Petrik (1958); Vorgin (1955); Lebedev (1931); Mocsáry (1897); Apfelbeck (1896). [DD]
61. ⸗ Andrena nasuta Giraud, 1863 in Mudri-Stojnić (2018); Markov (2017); Markov et al. (2016); Mudri-Stojnić et al. (2012); Vorgin (1918); Mocsáry (1897). [DD]
62. Andrena nigroaenea (Kirby, 1802) in Kuhlmann et al. (2020); Vorgin (1955). [LC]
63. ⸗ Andrena nitida (Müller, 1776) in Kuhlmann et al. (2020); Vorgin (1955); Mocsáry (1897); AĐ coll. [LC]
64. ⸗ Andrena nitidiuscula Schenck, 1853 in Kuhlmann et al. (2020); Mudri-Stojnić (2018); Markov et al. (2016); Mudri-Stojnić et al. (2012); Mocsáry (1897); as Andrena lucens Imhoff, 1868 in Vorgin (1955, 1918); Korlević (1890). [LC]
65. ▪ Andrena niveata Friese, 1887 in Kuhlmann et al. (2020). [DD]
66. Andrena nobilis Morawitz, 1874 in Kuhlmann et al. (2020); Vorgin (1955). [DD]
67. ⸗ Andrena oralis Morawitz, 1876 in Markov (2017); Markov et al. (2016); Vorgin (1955). [DD]
68. ⸗ Andrena ovatula (Kirby, 1802) in Kuhlmann et al. (2020); Mudri-Stojnić (2018); Markov (2017); Markov et al. (2016); Mudri-Stojnić et al. (2012); Živojinović (1950); AZ coll.; also as Andrena albofasciata Thomson, 1871 in Lebedev (1931); as Andrena afzeliella (Kirby, 1802) in Petrik (1958). [NT]
69. ⸗ Andrena pandellei Pérez, 1895 New material examined: 1 ♂; Novi Sad, Kamenički park; 45.2299°N, 19.8518°E; 20 Jun. 2018; A. Đukić leg.; Zsolt Józan det.; AĐ coll. [LC]
70. ⸗ Andrena paucisquama Noskiewicz, 1924 New material examined: 1 ♀; Fruška gora, Manastir Grgeteg; 45.1383°N, 19.9044°E; 20 May 2018; S. Mudri-Stojnić leg.; Zsolt Józan det.; FSUNS. [DD]
71. ⸗ Andrena pilipes Fabricius, 1781 in Kuhlmann et al. (2020); Petrik (1958); Vorgin (1918); Korlević (1890); ZM coll.; as Andrena carbonaria (Linnaeus, 1767) in Vorgin (1955); Lebedev (1931); Mocsáry (1897); Apfelbeck (1896). [LC]
72. ⸗ Andrena polita Smith, 1847 in Kuhlmann et al. (2020); Mudri-Stojnić (2018); Markov (2017); Markov et al. (2016); Mudri-Stojnić et al. (2012); Lebedev (1931). [LC]
73. ⸗ Andrena potentillae Panzer, 1809 in ZM coll. [DD]
74. ⸗ Andrena propinqua Schenck, 1853 in Kuhlmann et al. (2020); Lebedev (1931); Mocsáry (1897); ZM coll. [DD]
75. ⸗ Andrena proxima (Kirby, 1802) in Kuhlmann et al. (2020); Markov (2017); Markov et al. (2016); Vorgin (1955); Živojinović (1950); Lebedev (1931); Apfelbeck (1896). [DD]
* Andrena pyropygia Kriechbaumer, 1873 in Mocsáry (1897). [LC]
77. ⸗ Andrena rhenana Stoeckhert, 1930 in ZM coll. [DD]
78. ⸗ Andrena rosae Panzer, 1801 in Kuhlmann et al. (2020); Mudri-Stojnić (2018); Markov (2017); Markov et al. (2016); Mudri-Stojnić et al. (2012); Lebedev (1931). [DD]
79. ▪ Andrena rufula Schmiedeknecht, 1883 in Kuhlmann et al. (2020). [LC]
80. ▪ Andrena schencki Morawitz, 1866 in Kuhlmann et al. (2020). [DD]
81. ▪ Andrena schlettereri Friese, 1896 in Kuhlmann et al. (2020). [DD]
82. Andrena scita Eversmann, 1852 in Kuhlmann et al. (2020); Vorgin (1955, 1918); Mocsáry (1897); Apfelbeck (1896). [DD]
83. ⸗ Andrena seminuda Friese, 1896 in Kuhlmann et al. (2020); Markov (2017); Markov et al. (2016); as Andrena setigera Alfken, 1911 in Vorgin (1955). [DD]
84. Andrena sericata Imhoff, 1868 in Kuhlmann et al. (2020); Apfelbeck (1896). [DD]
85. ⸗ Andrena simillima Smith, 1851 New material examined: 1 ♀; Vlasina, Delnice-Ljote; 42.6933°N, 22.3176°E; 22 Jul. 2019; A. Đukić leg.; Andrej Gogala det.; AĐ coll. [LC]
86. ⸗ Andrena simontornyella Noskiewicz, 1939 in Kuhlmann et al. (2020); Markov (2017); Markov et al. (2016). [LC]
87. ⸗ Andrena subopaca Nylander, 1848 in Lebedev (1931); AĐ coll. [LC]
88. ⸗ Andrena suerinensis Perkins 1914 in ZM coll. [DD]
89. ⸗ Andrena symphyti Schmiedeknecht, 1883 in Markov (2017); Markov et al. (2016); AZ coll.; ZM coll. [DD]
90. ⸗ Andrena taraxaci Giraud, 1861 in Kuhlmann et al. (2020); Mudri-Stojnić (2018); Schwenninger (2015); Grozdanić (1971b); Grozdanić and Vasić (1965c); Vorgin (1955); Lebedev (1931); ZM coll. [DD]
91. ⸗ Andrena tarsata Nylander, 1848 in Vorgin (1918); ZM coll. [DD]
92. ⸗ Andrena thoracica (Fabricius, 1775) in Mudri-Stojnić (2018); Vorgin (1955); Lebedev (1931); Mocsáry (1897); Apfelbeck (1896). [DD]
93. ⸗ Andrena tibialis (Kirby, 1802) in Kuhlmann et al. (2020); Mudri-Stojnić (2018); Vorgin (1955); Apfelbeck (1896). [LC]
94. ⸗ Andrena trimmerana (Kirby, 1802) in Lebedev (1931); ZM coll. [DD]
95. Andrena truncatilabris Morawitz, 1877 in Kuhlmann et al. (2020); Vorgin (1955, 1918); Lebedev (1931); Mocsáry (1897); Apfelbeck (1896). [DD]
96. ⸗ Andrena ungeri Mavromoustakis, 1952 in Kuhlmann et al. (2020); Mudri-Stojnić (2018); Markov (2017); Mudri-Stojnić et al. (2012). [LC]
97. ⸗ Andrena vaga Panzer, 1799 in Lebedev (1931); ZM coll. [LC]
98. ⸗ Andrena variabilis Smith, 1853 in Kuhlmann et al. (2020); Markov (2017); Markov et al. (2016); Vorgin (1955); Lebedev (1931); AZ coll. [DD]
99. Andrena varians (Kirby, 1802) in Lebedev (1931); Vorgin (1955). [LC]
100. Andrena ventralis Imhoff, 1832 in Kuhlmann et al. (2020); Lebedev (1931). [DD]
101. ⸗ Andrena ventricosa Dours, 1873 in Markov (2017); Markov et al. (2016); Vorgin (1918). [DD]
102. ⸗ Andrena viridescens Viereck, 1916 in Lebedev (1931); ZM coll. [DD]
103. Andrena wilkella (Kirby, 1802) in Vorgin (1955); as Andrena convexiuscula Kirby, 1802 in Petrik (1958); Mocsáry (1897); Apfelbeck (1896). [DD]

*Camptopoeum* Spinola, 1843 (2 species)

1. ⸗ Camptopoeum frontale Fabricius 1804 in Kuhlmann et al. (2020); Mudri-Stojnić (2018); Mudri-Stojnić et al. (2012); Rafajlović and Seleši (1958); Vorgin (1918); Mocsáry (1897). [DD]
2. Camptopoeum friesei Mocsáry 1894 in Grozdanić (1971b); Rafajlović and Seleši (1958); Živojinović (1950); Mocsáry (1897). [LC]

*Clavipanurgus* Warncke, 1972 (1 species)

1. ▪ Clavipanurgus sculpturatus Morawitz 1872 in Kuhlmann et al. (2020). [DD]

*Melitturga* Latreille, 1809 (1 species)

1. ⸗ Melitturga clavicornis Latreille 1808 in Kuhlmann et al. (2020); Mudri-Stojnić (2018); Markov (2017); Markov et al. (2016); Mudri-Stojnić et al. (2012); Petrik (1958); Mocsáry (1897). [NT]

*Panurginus* Nylander, 1848 (2 species)

1. Panurginus labiatus Eversmann 1852 in Kuhlmann et al. (2020); Lebedev (1931). [DD]
2. ⸗ Panurginus herzi Morawitz, 1892 New material examined: 1 ♀; Vlasina, Vrtop; 42.7904°N, 22.372°E; 20 Jul. 2019; A. Đukić leg.; Andrej Gogala det.; AĐ coll. 1 ♀; Vlasina Rid; 42.7253°N, 22.3284°E; 22–23 Jul. 2019; A. Đukić leg.; Andrej Gogala det.; AĐ coll. [DD]

*Panurgus* Panzer, 1806 (2 species)

1. ⸗ Panurgus banksianus Kirby 1802 in Kuhlmann et al. (2020); AĐ coll.; ZM coll. [LC]
2. ⸗ Panurgus calcaratus Scopoli, 1763 in Kuhlmann et al. (2020); Mudri-Stojnić (2018); Markov (2017); Markov et al. (2016); Rafajlović and Seleši (1958); Živojinović (1950); Lebedev (1931); Mocsáry (1897); Apfelbeck (1896); AĐ coll.; as Panurgus lobatus (Panzer, 1799) in Petrik (1958). [LC]

### Apidae (18 genera; 226 species)

*Amegilla* Friese, 1897 (5 species)

1. ⸗ Amegilla albigena Lepeletier, 1841 in Kuhlmann et al. (2020); ZM coll.; as Anthophora albigena Lepeletier, 1841 in Rafajlović and Seleši (1958); Vorgin (1955, 1918); Mocsáry (1897). [LC]
2. ⸗ Amegilla garrula Rossi, 1790 in Kuhlmann et al. (2020); Markov (2017); Markov et al. (2016); as Anthophora garrula Rossi, 1790 in Rafajlović and Seleši (1958); Živojinović (1950); as Anthophora garrulus in Mocsáry (1897). [LC]
3. Amegilla magnilabris (Fedtschenko 1875) as Anthophora magnilabris Fedtschenko, 1875 in Rafajlović and Seleši (1958); Mocsáry (1897). [DD]
4. ⸗ Amegilla quadrifasciata de Villers, 1789 in Kuhlmann et al. (2020); Mudri-Stojnić (2018); Mudri-Stojnić et al. (2012); Stanisavljević (2000); ZM coll.; as Anthophora quadrifasciata (de Villers, 1789) in Petrik (1958); Rafajlović and Seleši (1958); Lebedev (1931); as Anthophora quadrifasciatus in Mocsáry (1897). [DD]
5. ⸗ Amegilla salviae Morawitz, 1876 in Kuhlmann et al. (2020); ZM coll. [DD]

*Ammobatoides* Radoszkowski, 1867 (1 species)

1. ⸗ Ammobatoides abdominalis (Eversmann, 1852) in ZM coll. [EN]

*Ammobates* Latreille, 1809 (2 species)

* Ammobates punctatus (Fabricius, 1804) in Mocsáry (1897). [LC]
* Ammobates vinctus Gerstäcker, 1869 in Mocsáry (1897). [LC]

*Anthophora* Latreille, 1803 (18 species)

1. Anthophora aestivalis Panzer 1801 in Kuhlmann et al. (2020); Lebedev (1931). [LC]
2. ▪ Anthophora atroalba Lepeletier 1841 in Kuhlmann et al. (2020). [DD]
3. ⸗ Anthophora bimaculata Panzer, 1798 in Kuhlmann et al. (2020); Rafajlović and Seleši (1958); Lebedev (1931); Vorgin (1918); ZM coll.; as Saropoda bimaculata in Petrik (1958); as Anthophora bimaculatus in Mocsáry (1897). [LC]
4. ▪ Anthophora canescens Brullé 1832 in Kuhlmann et al. (2020). [DD]
5. Anthophora crassipes Lepeletier, 1841 in Lebedev (1931). [DD]
6. ▪ Anthophora dalmatica Pérez 1902 in Kuhlmann et al. (2020). [DD]
7. ▪ Anthophora dufourii Lepeletier 1841 in Kuhlmann et al. (2020). [DD]
8. ⸗ Anthophora fulvitarsis Brullé 1832 in ZM coll. [DD]
9. ⸗ Anthophora furcata Panzer, 1798 in Kuhlmann et al. (2020); Markov (2017); Markov et al. (2016); Rafajlović and Seleši (1958); Lebedev (1931); AĐ coll.; ZM coll.; as Anthophora furcatus in Mocsáry (1897). [LC]
10. ▪ Anthophora orientalis Morawitz 1877 in Kuhlmann et al. (2020). [DD]
11. ⸗ Anthophora plagiata Illiger, 1806 in Kuhlmann et al. (2020); as Anthophora parietina (Fabricius, 1793) in Mučalica (1987a, 1987b); Grozdanić (1971b); Grozdanić and Stevanović (1965); Grozdanić and Vasić (1965b); Rafajlović and Seleši (1958); Vorgin (1918); ZM coll.; Anthophora parietinus in Mocsáry (1897). [LC]
12. ⸗ Anthophora plumipes Pallas, 1772 in Kuhlmann et al. (2020); Markov (2017); Markov et al. (2016); AĐ coll.; ZM coll.; as Anthophora acervorum (Linnaeus, 1758) in Grozdanić (1971b); Grozdanić and Vasić (1965b, 1965c); Rafajlović and Seleši (1958); Živojinović (1950); Lebedev (1931). [LC]
13. Anthophora podagra Lepeletier, 1841 in Kuhlmann et al. (2020); as Anthophora podagrus in Mocsáry (1897). [DD]
14. ⸗ Anthophora pubescens Fabricius 1781 in Kuhlmann et al. (2020); Grozdanić and Radivojević (1972); Grozdanić (1971b); Rafajlović and Seleši (1958); Lebedev (1931); Mocsáry (1897); SG coll. [DD]
15. Anthophora quadrimaculata Panzer, 1798 in Kuhlmann et al. (2020); as Anthophora vulpina (Panzer, 1798) in Rafajlović and Seleši (1958); as Anthophora vulpinusMocsáry (1897). [DD]
16. ⸗ Anthophora retusa Linnaeus, 1758 in Kuhlmann et al. (2020); Rafajlović and Seleši (1958); Lebedev (1931); ZM coll.; as Anthophora retusus in Mocsáry (1897). [LC]
17. ▪ Anthophora robusta Klug 1845 in Kuhlmann et al. (2020). [DD]
18. ⸗ Anthophora salviae (Panzer, 1804) in Kuhlmann et al. (2020); Vorgin (1955, 1918); Lebedev (1931); Mocsáry (1897); Korlević (1890); also as Anthophora crinipes Smith, 1854 in Rafajlović and Seleši (1958); as Anthophora crin ipes in Markov (2017); Markov et al. (2016); Mučalica (1997, 1990, 1987b); Grozdanić (1971b); Grozdanić and Mučalica (1969); Grozdanić and Vasić (1966a, 1965b); AĐ coll.; ZM coll. [DD]

*Apis* Linnaeus, 1768 (1 species)

1. ⸗ Apis mellifera Linnaeus 1758 in Kuhlmann et al. (2020); Mudri-Stojnić (2018); Markov (2017); Markov et al. (2016); Mudri-Stojnić et al. (2012); Kulinčević et al. (1997); Krunić (1994, 1986); Grozdanić (1970, 1958b); Grozdanić and Vasić (1966a); Petrik (1958); Vlatković (1957); Živojinović (1950); Mocsáry (1897); Apfelbeck (1896); AĐ coll.; ZM coll. [DD]

*Biastes* Panzer, 1806 (3 species)

1. ⸗ Biastes brevicornis (Panzer, 1798) in Petrik (1958); Rafajlović and Seleši (1958); Mocsáry (1897); Apfelbeck (1896); SG coll. [LC]
2. Biastes emarginatus (Schenck, 1853) in Vorgin (1918). [LC]
3. ▪ Biastes truncatus (Nylander, 1848) in Kuhlmann et al. (2020). [VU]

*Bombus* Latreille, 1802 (47 species)

1. ⸗ Bombus argillaceus (Scopoli, 1763) in Kuhlmann et al. (2020); Mudri-Stojnić (2018); Markov (2017); Markov et al. (2016); Rasmont et al. (2015); Grozdanić (1971b, 1970); Grozdanić and Vasić (1965c); Grozdanić and Čolović (1955b); Lebedev (1931); ZM coll. [LC]
2. ▪ Bombus armeniacus Radoszkowski, 1877 in Kuhlmann et al. (2020). [EN]
3. ⸗ Bombus barbutellus Kirby, 1802 in Kuhlmann et al. (2020); Rasmont et al. (2015); SG coll.; as Psithyrus barbutellus (Kirby, 1802) in Grozdanić and Vasić (1966a); Vorgin (1955); Mocsáry (1897). [LC]
4. Bombus bohemicus Seidl, 1838 in Kuhlmann et al. (2020); Rasmont et al. (2015). [LC]
5. ⸗ Bombus campestris Panzer, 1801 in Kuhlmann et al. (2020); Rasmont et al. (2015); AĐ coll; as Psithyrus campestris f. francisanus K. and also as Psithyrus campestris f. rosiellus K. in Vorgin (1955). [LC]
6. Bombus confusus Schenck, 1861 in Kuhlmann et al. (2020); Rasmont et al. (2015); Mocsáry (1897). [VU]
7. ▪ Bombus cryptarum Fabricius, 1775 in Kuhlmann et al. (2020). [LC]
8. Bombus cullumanus Kirby, 1802 in Kuhlmann et al. (2020); as Bombus serrisquama Morawitz, 1888 in Lebedev (1931). [CR]
9. Bombus deuteronymus Schulz, 1879 in Kuhlmann et al. (2020); Rasmont et al. (2015); as Bombus bureschi Pittioni, 1939 in Živojinović (1950). [DD]
10. ▪ Bombus distinguendus Morawitz, 1869 in Kuhlmann et al. (2020). [VU]
11. ▪ Bombus flavidus Eversmann, 1852 in Kuhlmann et al. (2020). [LC]
12. Bombus fragrans (Pallas, 1771) in Kuhlmann et al. (2020); Mocsáry (1897). [EN]
13. ▪ Bombus gerstaeckeri Morawitz, 1881 in Kuhlmann et al. (2020). [VU]
14. ⸗ Bombus haematurus Kriechbaumer, 1870 in Kuhlmann et al. (2020); Markov (2017); Markov et al. (2016); Rasmont et al. (2015); Grozdanić (1971b); Grozdanić and Vasić (1965c); Živojinović (1950); Lebedev (1931); ZM coll. [LC]
15. ⸗ Bombus hortorum Linnaeus, 1761 in Kuhlmann et al. (2020); Markov (2017); Markov et al. (2016); Rasmont et al. (2015); Grozdanić (1971b, 1970); Grozdanić and Vasić (1966a); Grozdanić and Vasić (1965c); Grozdanić and Baranov (1963); Vorgin (1955); Živojinović (1950); Lebedev (1931); Apfelbeck (1896); AĐ coll.; ZM coll. [LC]
16. ⸗ Bombus humilis Illiger, 1806 in Kuhlmann et al. (2020); Mudri-Stojnić (2018); Markov (2017); Rasmont et al. (2015); Mudri-Stojnić et al. (2012); Grozdanić and Vasić (1966a, 1965c); Živojinović (1950); AĐ coll.; ZM coll.; as Bombus helferanus Seidl, 1838 in Vorgin (1955); Lebedev (1931); as Bombus variabilis Schmiedeknecht, 1878 in Mocsáry (1897); Apfelbeck (1896). [LC]
17. ⸗ Bombus hypnorum Linnaeus, 1758 in Kuhlmann et al. (2020); Markov (2017); Rasmont et al. (2015); Mudri-Stojnić et al. (2012); Živojinović (1950); Lebedev (1931); AĐ coll. [LC]
18. ▪ Bombus jonellus Kirby, 1802 in Kuhlmann et al. (2020). [LC]
19. Bombus laesus Morawitz, 1875 in Kuhlmann et al. (2020); Vorgin (1918); Mocsáry (1897). [NT]
20. ⸗ Bombus lapidarius Linnaeus, 1758 in Kuhlmann et al. (2020); Mudri-Stojnić (2018); Markov (2017); Rasmont et al. (2015); Mudri-Stojnić et al. (2012); Grozdanić (1971b, 1970); Grozdanić and Vasić (1966a, 1965c); Vorgin (1955); Živojinović (1950); Lebedev (1931); Mocsáry (1897); Apfelbeck (1896); AĐ coll.; ZM coll. [LC]
21. ⸗ Bombus lucorum Linnaeus, 1761 in Kuhlmann et al. (2020); Vorgin (1955); Živojinović (1950); Lebedev (1931); AĐ coll.; ZM coll. [LC]
22. ▪ Bombus mendax Gerstäcker, 1869 in Kuhlmann et al. (2020). [NT]
23. ▪ Bombus mesomelas Gerstäcker, 1869 in Kuhlmann et al. (2020). [LC]
24. ▪ Bombus mlokosievitzii Radoszkowski, 1877 in Kuhlmann et al. (2020). [DD]
25. ▪ Bombus mocsaryi Kriechbaumer, 1877 in Kuhlmann et al. (2020). [EN]
26. ▪ Bombus monticola Smith, 1849 in Kuhlmann et al. (2020). [LC]
27. ▪ Bombus mucidus Gerstäcker, 1869 in Kuhlmann et al. (2020). [NT]
28. Bombus muscorum Linnaeus, 1758 in Kuhlmann et al. (2020); Stevanović and Lazarov (1977); Vorgin (1955). [VU]
29. Bombus niveatus Kriechbaumer, 1870 in Kuhlmann et al. (2020); Rasmont et al. (2015); as Bombus vorticosus Gerstäcker, 1872 in Vorgin (1955). [LC]
30. ▪ Bombus norvegicus Sparre-Schneider, 1918 in Kuhlmann et al. (2020). [LC]
31. ⸗ Bombus pascuorum Scopoli, 1763 in Kuhlmann et al. (2020); Mudri-Stojnić (2018); Markov (2017); Markov et al. (2016); Rasmont et al. (2015); Mudri-Stojnić et al. (2012); AĐ coll.; ZM coll.; as Bombus cognatus Stephens, 1846 in Grozdanić (1971b); Grozdanić and Vasić (1965c); Lebedev (1931); Vorgin (1918); as Bombus agrorum (Fabricius, 1787) in Grozdanić and Vasić (1966a, 1965c); Grozdanić (1960); Grozdanić and Stevanović (1959); Petrik (1958); Grozdanić and Čolović (1955a, 1955b); Vorgin (1955); Živojinović (1950); Lebedev (1931); Mocsáry (1897); Apfelbeck (1896); Korlević (1890). [LC]
32. ⸗ Bombus pomorum Panzer, 1805 in Kuhlmann et al. (2020); Rasmont et al. (2015); Vorgin (1918); Mocsáry (1897); AĐ coll. [VU]
33. ⸗ Bombus pratorum Linnaeus, 1761 in Kuhlmann et al. (2020); Markov (2017); Rasmont et al. (2015); Grozdanić and Čolović (1955a, 1955b); Živojinović (1950); Lebedev (1931); Mocsáry (1897); AĐ coll. [LC]
34. Bombus pyrenaeus Pérez, 1879 in Kuhlmann et al. (2020); Rasmont et al. (2015). [LC]
35. ⸗ Bombus quadricolor Lepeletier, 1832 in Kuhlmann et al. (2020); Rasmont et al. (2015); AĐ coll. [LC]
36. ⸗ Bombus ruderarius Müller, 1776 in Kuhlmann et al. (2020); Mudri-Stojnić (2018); Markov (2017); Rasmont et al. (2015); Mudri-Stojnić et al. (2012); AĐ coll.; as Bombus derhamellus (Kirby, 1802) in Lebedev (1931); Apfelbeck (1896). [LC]
37. Bombus ruderatus Fabricius, 1775 in Petrik (1958); Vorgin (1955). [LC]
38. ⸗ Bombus rupestris Fabricius, 1793 in Kuhlmann et al. (2020); Rasmont et al. (2015); AĐ coll.; ZM coll. [LC]
39. ▪ Bombus sichelii Radoszkowski, 1859 in Kuhlmann et al. (2020). [LC]
40. ⸗ Bombus soroeensis Fabricius, 1776 in Kuhlmann et al. (2020); Rasmont et al. (2015); Vorgin (1955); ZM coll.; as Bombus proteus Gerstäcker, 1869 in Lebedev (1931). [LC]
41. Bombus subterraneus (Linnaeus, 1758) in Kuhlmann et al. (2020); Rasmont et al. (2015). [LC]
42. ⸗ Bombus sylvarum Linnaeus, 1761 in Kuhlmann et al. (2020); Mudri-Stojnić (2018); Markov (2017); Rasmont et al. (2015); Mudri-Stojnić et al. (2012); Grozdanić (1971b); Grozdanić and Vasić (1966a, 1965c); Vorgin (1955); Živojinović (1950); Lebedev (1931); Mocsáry (1897); Apfelbeck (1896); Korlević (1890); SG coll. [LC]
43. ⸗ Bombus sylvestris Lepeletier, 1832 in Kuhlmann et al. (2020); Rasmont et al. (2015); AĐ coll. [LC]
44. ⸗ Bombus terrestris Linnaeus, 1758 in Kuhlmann et al. (2020); Mudri-Stojnić (2018); Markov (2017); Markov et al. (2016); Rasmont et al. (2015); Mudri-Stojnić et al. (2012); Grozdanić (1971b, 1970); Grozdanić and Vasić (1966a, 1965c); Grozdanić and Baranov (1963); Petrik (1958); Grozdanić and Čolović (1955b); Vorgin (1955); Živojinović (1950); Lebedev (1931); Apfelbeck (1896); AĐ coll.; ZM coll. [LC]
45. ⸗ Bombus vestalis Geoffroy, 1785 in Kuhlmann et al. (2020); Rasmont et al. (2015); AĐ coll.; SG coll.; as Psithyrus vestalis Geoffroy, 1785 in Grozdanić and Vasić (1966a); Vorgin (1955); Lebedev (1931); Apfelbeck (1896). [LC]
46. ⸗ Bombus wurflenii Radoszkowski, 1859 in Kuhlmann et al. (2020); Rasmont et al. (2015); AĐ coll.; as Bombus mastrucatus Gerstäcker, 1869 in Apfelbeck (1896). [LC]
47. Bombus zonatus Smith, 1854 in Kuhlmann et al. (2020); Živojinović (1950); Lebedev (1931); Mocsáry (1897). [EN]

*Ceratina* Latreille, 1802 (11 species)

1. ⸗ Ceratina acuta Friese, 1896 in Grozdanić (1971b); Rafajlović and Seleši (1958); ZM coll. [LC]
2. ⸗ Ceratina callosa Fabricius, 1794 in Grozdanić (1971b); Rafajlović and Seleši (1958); Živojinović (1950); Lebedev (1931); Mocsáry (1897); ZM coll. [LC]
3. ⸗ Ceratina chalcites Germar, 1839 in Kuhlmann et al. (2020); Markov (2017); Markov et al. (2016); Rafajlović and Seleši (1958); Lebedev (1931); Vorgin (1918); Mocsáry (1897). [LC]
4. ⸗ Ceratina chalybea Chevrier, 1872 in Kuhlmann et al. (2020); Markov (2017); Markov et al. (2016). [LC]
5. ⸗ Ceratina cucurbitina Rossi, 1792 in Kuhlmann et al. (2020); Markov (2017); Markov et al. (2016); Grozdanić (1971b); Rafajlović and Seleši (1958); Lebedev (1931); Mocsáry (1897); Apfelbeck (1896); ZM coll. [LC]
6. ⸗ Ceratina cyanea Kirby, 1802 in Kuhlmann et al. (2020); Markov (2017); Markov et al. (2016); Rafajlović and Seleši (1958); Lebedev (1931); Mocsáry (1897); Apfelbeck (1896); AĐ coll.; ZM coll. [LC]
7. ▪ Ceratina dallatorreana Friese, 1896 in Kuhlmann et al. (2020). [LC]
8. ▪ Ceratina dentiventris Gerstäcker, 1869 in Kuhlmann et al. (2020). [LC]
9. ⸗ Ceratina loewi Gerstäcker, 1869 in ZM coll. [DD]
10. Ceratina nigroaenea Gerstäcker, 1869 in Rafajlović and Seleši (1958). [LC]
11. ⸗ Ceratina nigrolabiata Friese, 1896 in Kuhlmann et al. (2020); Mudri-Stojnić (2018); Markov (2017); Markov et al. (2016); Mudri-Stojnić et al. (2012); Grozdanić (1971b); Rafajlović and Seleši (1958); AĐ coll.; ZM coll. [LC]

*Epeoloides* Giraud, 1863 (1 species)

1. ⸗ Epeoloides coecutiens (Fabricius, 1775) New material examined: 1 ♂, 1 ♀; Vlasina, Blato, Božički kanal; 42.6786°N, 22.3543°E; 23 Jul. 2019; T. Tot, N. Veljković leg.; Andrej Gogala det.; AĐ coll. [LC]

*Epeolus* Latreille, 1802 (5 species)

1. Epeolus cruciger Panzer, 1799 in Kuhlmann et al. (2020); Bogusch and Hadrava (2018); Rafajlović and Seleši (1958). [NT]
2. Epeolus fasciatus Friese, 1895 in Bogusch and Hadrava (2018); Rafajlović and Seleši (1958); Lebedev (1931); Mocsáry (1897). [DD]
3. ⸗ Epeolus schummeli Schilling, 1849 in SG coll. [NT]
4. Epeolus transitorius Eversmann, 1852 in Bogusch and Hadrava (2018); Petrik (1958); as Epeolus julliani Pérez, 1884 in Kuhlmann et al. (2020). Note: According to Bogusch and Hadrava (2018)E. julliani is syn. nov. under E. transitorius. [DD]
5. ⸗ Epeolus variegatus Linnaeus, 1758 in Kuhlmann et al. (2020); Bogusch and Hadrava (2018); Markov (2017); Markov et al. (2016); Petrik (1958); Apfelbeck (1896); also as Epeolus productus Thomson, 1870 in Mocsáry (1897). [LC]

*Eucera* Scopoli, 1770 (33 species)

1. Eucera alternans Brullé, 1832 in Kuhlmann et al. (2020); as Tetralonia ruficollis (Brullé, 1832) in Petrik (1958); as Eucera ruficollis (Brullé, 1832) in Vorgin (1918). [DD]
2. Eucera caspica Morawitz, 1873 in Vorgin (1918). [LC]
3. ⸗ Eucera chrysopyga Pérez, 1854 in Kuhlmann et al. (2020); Markov (2017); Markov et al. (2016); Rafajlović and Seleši (1958); Lebedev (1931); Vorgin (1918); Mocsáry (1897); Apfelbeck (1896). [LC]
4. ⸗ Eucera cineraria Eversmann, 1852 in Kuhlmann et al. (2020); Markov (2017); Markov et al. (2016). [LC]
5. ⸗ Eucera clypeata Erichson, 1835 in Kuhlmann et al. (2020); Markov (2017); Markov et al. (2016); Mudri-Stojnić (2018); Mudri-Stojnić et al. (2012); Lebedev (1931); Vorgin (1918); Mocsáry (1897); also as Eucera similis Lepeletier, 1841 in Rafajlović and Seleši (1958); Apfelbeck (1896). [LC]
6. Eucera dalmatica Lepeletier, 1841 in Kuhlmann et al. (2020); Lebedev (1931); Mocsáry (1897). [LC]
7. ⸗ Eucera excisa Mocsáry, 1879 in Grozdanić (1971b, 1969a); Grozdanić and Vasić (1967b); Vorgin (1918); Lebedev (1931); Mocsáry (1897); ZM coll. [DD]
8. ⸗ Eucera hungarica Friese, 1896 in Mocsáry (1897); SG coll.; also as Tetralonia hungarica (Friese, 1896) in Rafajlović and Seleši (1958). [LC]
9. ⸗ Eucera interrupta Bär, 1850 in Kuhlmann et al. (2020); Mudri-Stojnić (2018); Markov (2017); Markov et al. (2016); Rafajlović and Seleši (1958); Lebedev (1931); Mocsáry (1897). [LC]
10. ⸗ Eucera longicornis Linnaeus, 1758 in Kuhlmann et al. (2020); Mudri-Stojnić (2018); Petrik (1958); Rafajlović and Seleši (1958); Vorgin (1955); Živojinović (1950); Lebedev (1931); AĐ coll.; SG coll.; ZM coll.; also as Eucera difficilis Pérez, 1879 in Mocsáry (1897); Apfelbeck (1896). [LC]
11. ⸗ Eucera nigrescens Pérez, 1879 in Kuhlmann et al. (2020); Markov (2017); Markov et al. (2016); ZM coll.; as Eucera tuberculata (Fabricius, 1793) in Grozdanić and Vasić (1966a, 1965c); Rafajlović and Seleši (1958); Lebedev (1931). [LC]
12. ⸗ Eucera nigrifacies Lepeletier, 1841 in Kuhlmann et al. (2020); Markov (2017); Markov et al. (2016); Mocsáry (1897). [LC]
13. ⸗ Eucera pollinaris Kirby, 1802 in Markov (2017); Markov et al. (2016); as Eucera armeniaca (Morawitz, 1877) in Rafajlović and Seleši (1958). [DD]
14. Eucera proxima Morawitz, 1875 as Eucera nitidiventris Mocsáry, 1879 in Rafajlović and Seleši (1958); Vorgin (1918); Apfelbeck (1896). [DD]
15. ▪ Eucera punctulata Alfken, 1942 in Kuhlmann et al. (2020). [DD]
16. ⸗ Eucera seminuda Brullé, 1832 in Mudri-Stojnić (2018); Markov (2017); Markov et al. (2016); Lebedev (1931); Vorgin (1918); ZM coll. [LC]
17. ⸗ Eucera taurica Morawitz, 1871 in Kuhlmann et al. (2020); Markov (2017); Markov et al. (2016). [DD]
18. Eucera tricincta Erichson, 1835 in Vorgin (1918); also as Tetralonia tricincta (Erichson, 1835) in Rafajlović and Seleši (1958). [LC]
19. ▪ Eucera vittulata Noskiewicz, 1934 in Kuhlmann et al. (2020). [DD]
20. Eucera vulpes Brullé, 1832 in Kuhlmann et al. (2020); as Eucera parvula Friese, 1896 in Rafajlović and Seleši (1958). [DD]

Note: According to Dorchin et al. (2018), genera *Cubitalia* Friese, 1911, *Tetralonia* Spinola, 1838 and *Tetraloniella* Ashmead, 1899 are placed as subgenera within *Eucera* Scopoli, 1770 (and *Tetraloniella* is synonymised with *Tetralonia*):

1. ⸗ Eucera (Cubitalia) parvicornis Mocsáry, 1878 as Cubitalia parvicornis in ZM coll.; as Eucera parvicornis Mocsáry, 1878 in Rafajlović and Seleši (1958); Vorgin (1918); Mocsáry (1897). [DD]
2. ⸗ Eucera (Tetralonia) malvae Rossi, 1790 as Tetralonia malvae in Kuhlmann et al. (2020); Grozdanić (1971b); Vorgin (1955); Živojinović (1950); Lebedev (1931); ZM coll.; also as Eucera malvae (Rossi, 1790) in Rafajlović and Seleši (1958); as Eucera malvae in Mocsáry (1897); Apfelbeck (1896). [LC]
3. ⸗ Eucera (Tetralonia) alticincta Lepeletier, 1841 as Tetraloniella alticincta in Kuhlmann et al. (2020); Markov (2017); as Tetralonia alticincta (Lepeletier, 1841) in Mudri-Stojnić (2018); Mudri-Stojnić et al. (2012). [LC]
4. ⸗ Eucera (Tetralonia) dentata Germar, 1839 as Tetraloniella dentata in Kuhlmann et al. (2020); as Tetralonia dentata (Klug, 1835) in Mudri-Stojnić (2018); Lebedev (1931); as Eucera dentata Germar, 1839 in Rafajlović and Seleši (1958); Vorgin (1918); Mocsáry (1897). [LC]
5. Eucera (Tetralonia) fulvescens Giraud, 1863 as Tetraloniella fulvescens in Kuhlmann et al. (2020); as Tetralonia dufouri (Pérez, 1879) in Vorgin (1955). [DD]
6. ▪ Eucera (Tetralonia) glauca Fabricius 1775 as Tetraloniella glauca in Kuhlmann et al. (2020). [DD]
7. ⸗ Eucera (Tetralonia) graja (Eversmann, 1852) as Tetraloniella graja in Grozdanić (1971b); ZM coll. [DD]
8. ⸗ Eucera (Tetralonia) lyncea Mocsáry, 1879 as Tetraloniella lyncea in Markov (2017); as Tetralonia lyncea Mocsáry, 1879 in Mudri-Stojnić (2018); Mudri-Stojnić et al. (2012); Grozdanić (1971b); Grozdanić and Vasić (1966b); Lebedev (1931); SG coll.; ZM coll; as Eucera lyncea (Mocsáry, 1879) in Rafajlović and Seleši (1958); Mocsáry (1897). [DD]
9. ⸗ Eucera (Tetralonia) nana Morawitz, 1874 as Tetraloniella nana in Markov (2017); ZM coll.; as Tetralonia nana Morawitz, 1874 in Mudri-Stojnić (2018); Mudri-Stojnić et al. (2012); Grozdanić (1971b); Grozdanić and Vasić (1967a); Lebedev (1931); SG coll.; as Eucera nana (Morawitz, 1874) in Rafajlović and Seleši (1958); Vorgin (1918); Mocsáry (1897). [DD]
10. ⸗ Eucera (Tetralonia) ruficornis Fabricius, 1804 as Eucera ruficornis Fabricius, 1804 in Rafajlović and Seleši (1958); Mocsáry (1897); as Tetralonia ruficornis (Fabricius, 1804) in Vorgin (1955); Lebedev (1931); SG coll. [DD]
11. ⸗ Eucera (Tetralonia) pollinosa Lepeletier, 1841 as Tetralonia pollinosa (Lepeletier, 1841) and also as Eucera pollinosa Lepeletier, 1841 in Mudri-Stojnić (2018); as Eucera pollinosa in Rafajlović and Seleši (1958); Mocsáry (1897); as Macrocera fossulata in Korlević (1890). [DD]
12. ⸗ Eucera (Tetralonia) salicariae Lepeletier, 1841 as Eucera salicariae (Lepeletier, 1841) in Rafajlović and Seleši (1958); Vorgin (1918); Mocsáry (1897); Apfelbeck (1896); as Tetralonia salicariae (Lepeletier, 1841) in Vorgin (1955); Živojinović (1950); SG coll.; ZM coll. [DD]
13. ⸗ Eucera (Tetralonia) scabiosae Mocsáry, 1881 as Tetraloniella scabiosae in Kuhlmann et al. (2020); Markov (2017); as Tetralonia scabiosae (Mocsáry, 1881) in Mudri-Stojnić (2018); Mudri-Stojnić et al. (2012); Grozdanić (1971b); Rafajlović and Seleši (1958); SG coll. ZM coll.; as Eucera scabiosae Mocsáry, 1881 in Rafajlović and Seleši (1958); Vorgin (1955, 1918); Mocsáry (1897). [DD]

*Habropoda* Smith, 1854 (2 species)

1. ▪ Habropoda tarsata Spinola, 1838 in Kuhlmann et al. (2020). [LC]
2. ▪ Habropoda zonatula Smith, 1854 in Kuhlmann et al. (2020). [DD]

*Melecta* Latreille, 1802 (7 species)

1. ⸗ Melecta albifrons Förster, 1771 in Kuhlmann et al. (2020); as Melecta armata (Panzer, 1799) in Rafajlović and Seleši (1958); Vorgin (1918); ZM coll. [LC]
2. ▪ Melecta duodecimmaculata Rossi 1790 in Kuhlmann et al. (2020). [DD]
3. ▪ Melecta fulgida Lieftinck, 1980 in Kuhlmann et al. (2020). [DD]
4. ▪ Melecta funeraria Smith, 1854 in Kuhlmann et al. (2020). [DD]
5. ▪ Melecta italica Radoszkowski, 1876 in Kuhlmann et al. (2020). [DD]
6. ⸗ Melecta luctuosa Scopoli, 1770 in Kuhlmann et al. (2020); Rafajlović and Seleši (1958); Lebedev (1931); Vorgin (1918); ZM coll. [LC]
7. ▪ Melecta obscura Friese, 1895 in Kuhlmann et al. (2020). [DD]

*Nomada* Scopoli, 1770 (77 species)

1. Nomada alboguttata Herrich-Schäffer, 1839 in Kuhlmann et al. (2020); Smit (2018); Rafajlović and Seleši (1958); Lebedev (1931); Mocsáry (1897). [LC]
2. Nomada alpigena Schwarz, Gusenleitner, & Mazzucco, 1999 in Kuhlmann et al. (2020). Note: There are no type specimens listed from Serbia in Schwarz, Gusenleitner and Mazzucco (1999). [DD]
3. Nomada argentata Herrich-Schäffer, 1839 in Kuhlmann et al. (2020); Smit (2018). [NT]
4. ⸗ Nomada armata Herrich-Schäffer, 1839 in Kuhlmann et al. (2020); Smit (2018); Rafajlović and Seleši (1958); Mocsáry (1897); Apfelbeck (1896); AĐ coll. [NT]
5. Nomada atroscutellaris Strand, 1921 in Kuhlmann et al. (2020); Smit (2018). [LC]
6. Nomada basalis Herrich-Schäffer, 1839 in Kuhlmann et al. (2020); Smit (2018); Petrik (1958); Rafajlović and Seleši (1958); as Nomada tripunctata Morawitz, 1872 in Vorgin (1955, 1918); as Nomada flavomaculata Lucas, 1849 in Mocsáry (1897); Apfelbeck (1896). [LC]
7. ⸗ Nomada bifasciata Olivier, 1811 in Kuhlmann et al. (2020); Markov (2017); Markov et al. (2016); as Nomada pusilla in Rafajlović and Seleši (1958). [LC]
8. Nomada bispinosa Mocsáry, 1883 in Rafajlović and Seleši (1958); Lebedev (1931). [LC]
9. ⸗ Nomada bluethgeni Stoeckhert, 1943 in Kuhlmann et al. (2020); Smit (2018); Markov (2017); Markov et al. (2016). [LC]
10. Nomada braunsiana Schmiedeknecht, 1882 in Kuhlmann et al. (2020); Lebedev (1931). [NT]
* Nomada calimorpha Schmiedeknecht, 1882 in Mocsáry (1897). [DD]
12. ▪ Nomada castellana Dusmety Alonso, 1913 in Kuhlmann et al. (2020). [LC]
13. ▪ Nomada confinis Schmiedeknecht, 1882 in Kuhlmann et al. (2020). [DD]
14. Nomada conjungens Herrich-Schäffer, 1839 in Kuhlmann et al. (2020); Lebedev (1931). [LC]
15. Nomada cruenta Schmiedeknecht, 1882 in Kuhlmann et al. (2020); Smit (2018); Vorgin (1918). [LC]
16. ⸗ Nomada distinguenda Morawitz, 1874 in Smit (2018); Markov (2017); Markov et al. (2016); Rafajlović and Seleši (1958); Vorgin (1918); Apfelbeck (1896). [LC]
17. ▪ Nomada emarginata Morawitz, 1877 in Kuhlmann et al. (2020). [LC]
18. Nomada errans Lepeletier, 1841 in Kuhlmann et al. (2020); Smit (2018); Lebedev (1931). [NT]
19. Nomada erythrocephala Morawitz, 1870 in Rafajlović and Seleši (1958). [DD]
20. ⸗ Nomada fabriciana Linnaeus, 1767 in Kuhlmann et al. (2020); Lebedev (1931); AĐ coll. [LC]
21. ▪ Nomada facilis Schwarz, 1967 in Kuhlmann et al. (2020). [LC]
22. Nomada femoralis Morawitz, 1869 in Kuhlmann et al. (2020); Rafajlović and Seleši (1958); Vorgin (1918); Mocsáry (1897). [LC]
23. Nomada ferruginata Linné 1767 in Kuhlmann et al. (2020); Smit (2018); Rafajlović and Seleši (1958); Vorgin (1955, 1918); Lebedev (1931). [LC]
24. ⸗ Nomada flava Panzer, 1798 in Kuhlmann et al. (2020); Smit (2018); Lebedev (1931); AĐ coll. [LC]
25. ⸗ Nomada flavoguttata Kirby, 1802 in Kuhlmann et al. (2020); Smit (2018); Markov (2017); Markov et al. (2016); Rafajlović and Seleši (1958); Lebedev (1931); Apfelbeck (1896); AĐ coll. [LC]
26. ▪ Nomada flavopicta Kirby, 1802 in Kuhlmann et al. (2020). [LC]
27. ⸗ Nomada fucata Panzer, 1798 in Kuhlmann et al. (2020); Smit (2018); Rafajlović and Seleši (1958); Lebedev (1931); Vorgin (1918); Mocsáry (1897); Apfelbeck (1896); AĐ coll.; as Nomada varia Panzer, 1798 in Petrik (1958). [LC]
28. Nomada fulvicornis Fabricius, 1793 in Kuhlmann et al. (2020); Smit (2018); also as Nomada lineola Panzer, 1798 in Rafajlović and Seleši (1958); Lebedev (1931); as Nomada lineola in Vorgin (1955); Mocsáry (1897); as Nomada robusta Morawitz, 1870 in Apfelbeck (1896). [LC]
29. Nomada furva Panzer, 1798 in Kuhlmann et al. (2020); Rafajlović and Seleši (1958). [DD]
30. ▪ Nomada furvoides Stoeckhert, 1944 in Kuhlmann et al. (2020). [DD]
31. Nomada fuscicornis Nylander, 1848 in Kuhlmann et al. (2020); Smit (2018); Vorgin (1918). [LC]
32. ▪ Nomada goodeniana Kirby, 1802 in Kuhlmann et al. (2020). [LC]
* Nomada gribodoi Schmiedeknecht, 1882 as Nomada elegans Mocsáry, 1897 in Mocsáry (1897). [DD]
34. Nomada guttulata Schenck, 1861 in Kuhlmann et al. (2020); Smit (2018); Vorgin (1918). [LC]
35. ▪ Nomada hirtipes Pérez, 1884 in Kuhlmann et al. (2020). [LC]
36. Nomada hungarica Dalla Torre & Friese, 1894 in Rafajlović and Seleši (1958). [DD]
37. Nomada immaculata Morawitz, 1874 in Vorgin (1918). [DD]
38. Nomada incisa Schmiedeknecht, 1882 in Kuhlmann et al. (2020); Smit (2018); Lebedev (1931). [DD]
39. ⸗ Nomada integra Brullé, 1832 in Kuhlmann et al. (2020); AĐ coll. [LC]
40. Nomada kohli Schmiedeknecht, 1882 in Kuhlmann et al. (2020); Smit (2018); Apfelbeck (1896). [LC]
41. ⸗ Nomada lathburiana Kirby, 1802 in Kuhlmann et al. (2020); SG coll. [LC]
42. ⸗ Nomada leucophthalma (Kirby, 1802) New material examined: 1 ♀; Vlasina, Veliki čemernik; 42.7368°N, 22.2723°E; 25 May 2019; M. Vujić leg.; Andrej Gogala det.; AĐ coll. [LC]
43. Nomada marshamella Kirby, 1802 in Kuhlmann et al. (2020); Smit (2018). [LC]
44. Nomada mauritanica Lepeletier, 1841 in Smit (2018); as Nomada chrysopyga Morawitz, 1871 in Rafajlović and Seleši (1958); Vorgin (1918); Mocsáry (1897); Apfelbeck (1896). [LC]
45. Nomada mocsaryi Schmiedeknecht, 1882 in Kuhlmann et al. (2020); Smit (2018); Mocsáry (1897). [DD]
46. ▪ Nomada moeschleri Alfken, 1913 in Kuhlmann et al. (2020). [LC]
47. Nomada mutabilis Morawitz, 1870 in Kuhlmann et al. (2020); Smit (2018); Vorgin (1918); Apfelbeck (1896). [LC]
48. Nomada mutica Morawitz, 1872 in Kuhlmann et al. (2020); Lebedev (1931). [NT]
49. Nomada nobilis Herrich-Schäffer, 1839 in Kuhlmann et al. (2020); Smit (2018); Mocsáry (1897); Apfelbeck (1896). [LC]
50. Nomada numida Lepeletier, 1841 in Kuhlmann et al. (2020); Smit (2018). [LC]
51. ▪ Nomada obtusifrons Nylander, 1848 in Kuhlmann et al. (2020). [NT]
52. ▪ Nomada opaca Alfken, 1913 in Kuhlmann et al. (2020). [NT]
53. ▪ Nomada pallispinosa Schwarz, 1967 in Kuhlmann et al. (2020). [DD]
54. Nomada panzeri Lepeletier, 1841 in Kuhlmann et al. (2020); Smit (2018). [LC]
55. Nomada pectoralis Morawitz, 1877 in Vorgin (1918). [DD]
56. Nomada piccioliana Magretti, 1883 in Kuhlmann et al. (2020); Smit (2018). [LC]
57. Nomada pleurosticta Herrich-Schäffer, 1839 as Nomada major Morawitz, 1872 in Lebedev (1931). [NT]
58. ▪ Nomada propinqua Schmiedeknecht, 1882 in Kuhlmann et al. (2020). [LC]
59. Nomada pulchra Arnold, 1888 in Smit (2018). [EN]
60. Nomada rhenana Morawitz, 1872 in Kuhlmann et al. (2020); Smit (2018); Rafajlović and Seleši (1958); Mocsáry (1897). [NT]
61. ⸗ Nomada ruficornis Linnaeus, 1758 in Kuhlmann et al. (2020); Smit (2018); Apfelbeck (1896); Mocsáry (1897); AĐ coll; also as Nomada bifida Thomson, 1872 in Rafajlović and Seleši (1958); as Nomada bifida in Lebedev (1931). [LC]
62. Nomada rufipes Fabricius, 1793 in Kuhlmann et al. (2020); Petrik (1958); Rafajlović and Seleši (1958). [LC]
63. Nomada sexfasciata Panzer, 1799 in Kuhlmann et al. (2020); Smit (2018); Rafajlović and Seleši (1958); Lebedev (1931). [LC]
64. ▪ Nomada sheppardana Kirby, 1802 in Kuhlmann et al. (2020). [LC]
65. Nomada signata Jurine, 1807 in Kuhlmann et al. (2020); Rafajlović and Seleši (1958). [LC]
66. Nomada similis Morawitz, 1872 in Vorgin (1918). [LC]
67. Nomada stigma Fabricius, 1804 in Kuhlmann et al. (2020); as Nomada austriaca Schmiedeknecht, 1882 in Vorgin (1918). [LC]
68. Nomada striata Fabricius, 1793 in Kuhlmann et al. (2020); as Nomada ochrostoma (Kirby, 1802) in Apfelbeck (1896). [LC]
69. ▪ Nomada succincta Panzer, 1798 in Kuhlmann et al. (2020). [LC]
70. ▪ Nomada symphyti Stoeckhert, 1930 in Kuhlmann et al. (2020). [NT]
71. Nomada tenella Mocsáry, 1883 in Kuhlmann et al. (2020); Smit (2018). [NT]
72. Nomada transitoria Schmiedeknecht, 1882 in Smit (2018). [LC]
73. ▪ Nomada tridentirostris Dours, 1873 in Kuhlmann et al. (2020). [LC]
74. Nomada trispinosa Schmiedeknecht, 1882 in Kuhlmann et al. (2020); Rafajlović and Seleši (1958); Lebedev (1931). [LC]
75. ▪ Nomada verna Schmiedeknecht, 1882 in Kuhlmann et al. (2020). [DD]
76. Nomada villosa Thomson, 1870 in Kuhlmann et al. (2020); Lebedev (1931). [NT]
77. Nomada zonata Panzer, 1798 in Kuhlmann et al. (2020); Smit (2018); Lebedev (1931); Vorgin (1918). [LC]

*Pasites* Jurine, 1807 (1 species)

1. Pasites maculatus Jurine, 1807 in Petrik (1958); Rafajlović and Seleši (1958); Mocsáry (1897). [LC]

*Thyreus* Panzer, 1806 (7 species)

1. Thyreus affinis Morawitz, 1874 in Kuhlmann et al. (2020); as Crocisa affinis Morawitz, 1874 in Rafajlović and Seleši (1958). [DD]
2. ⸗ Thyreus histrionicus Illiger, 1806 in Kuhlmann et al. (2020); ZM coll.; as Crocisa major Morawitz, 1875 in Rafajlović and Seleši (1958); Mocsáry (1897). [LC]
3. ⸗ Thyreus orbatus Lepeletier, 1841 in Kuhlmann et al. (2020); ZM coll. [LC]
4. ▪ Thyreus picaron Lieftinck, 1968 in Kuhlmann et al. (2020). [DD]
5. ⸗ Thyreus ramosus Lepeletier, 1841 in Kuhlmann et al. (2020); ZM coll.; as Crocisa ramosa Lepeletier, 1841 in Rafajlović and Seleši (1958); Mocsáry (1897). [LC]
6. ⸗ Thyreus scutellaris Fabricius, 1781 in Kuhlmann et al. (2020); ZM coll.; as Crocisa scutellaris (Fabricius, 1781) in Petrik (1958); Vorgin (1918); Mocsáry (1897). [DD]
7. Thyreus truncatus Pérez, 1883 in Kuhlmann et al. (2020); as Crocisa truncata Pérez, 1883 in Mocsáry (1897). [DD]

*Triepeolus* Robertson, 1901 (1 species)

* Triepeolus tristis (Smith, 1854) as Epeolus tristis Smith, 1854 in Mocsáry (1897). [NT]

*Xylocopa* Latreille, 1802 (4 species)

1. ⸗ Xylocopa iris Christ, 1791 in Kuhlmann et al. (2020); ZM coll.; as Xylocopa cyanescens Brullé, 1832 in Grozdanić and Mučalica (1973); Grozdanić (1971b); Grozdanić and Vasić (1966a); Lebedev (1931). [LC]
2. ▪ Xylocopa olivieri Lepeletier, 1841 in Kuhlmann et al. (2020). [LC]
3. ⸗ Xylocopa valga Gerstäcker, 1872 in Kuhlmann et al. (2020); Grozdanić and Mučalica (1973); Grozdanić (1971b, 1970, 1950b); Grozdanić and Vasić (1965c); Grozdanić and Baranov (1963); Rafajlović and Seleši (1958); Grozdanić and Čolović (1955b); Mocsáry (1897); Apfelbeck (1896); AĐ coll.; ZM coll. [LC]
4. ⸗ Xylocopa violacea Linnaeus, 1758 in Kuhlmann et al. (2020); Grozdanić and Mučalica (1973); Grozdanić (1971b, 1950b); Grozdanić and Vasić (1966a); Petrik (1958); Rafajlović and Seleši (1958); Živojinović (1950); Lebedev (1931); Apfelbeck (1896); AĐ coll.; ZM coll. [LC]

### Colletidae (2 genera; 69 species)

*Colletes* Latreille, 1802 (27 species)

1. ▪ Colletes albomaculatus (Lucas, 1849) in Kuhlmann et al. (2020). [NT]
2. ⸗ Colletes anchusae Noskiewicz, 1924 Kuhlmann et al. (2020); Grozdanić and Vasić (1966a); ZM coll. [EN]
3. Colletes brevigena Noskiewicz, 1936 in Kuhlmann et al. (2020); Burger (2010). [LC]
4. ▪ Colletes carinatus Radoszkowski, 1891 in Kuhlmann et al. (2020). [LC]
5. ▪ Colletes caskanus (Strand, 1919) in Kuhlmann et al. (2020). [DD]
6. ▪ Colletes chengtehensis Yasumatsu, 1935 in Kuhlmann et al. (2020). [VU]
7. ⸗ Colletes cunicularius (Linnaeus, 1761) in Kuhlmann et al. (2020); Markov (2017); Markov et al. (2016); Grozdanić (1971b, 1958a); Rafajlović and Seleši (1958); Mocsáry (1897); AĐ coll.; ZM coll.; as Colletes cunicularia in Apfelbeck (1896). [LC]
8. ⸗ Colletes daviesanus Smith, 1846 in Kuhlmann et al. (2020); Markov (2017); Markov et al. (2016); Rafajlović and Seleši (1958); Lebedev (1931); Mocsáry (1897); AĐ coll.; as Colletes daviesana in Apfelbeck (1896). [LC]
9. Colletes eous Morice, 1904 in Kuhlmann et al. (2020); Lebedev (1931). [LC]
10. ▪ Colletes floralis Eversmann, 1852 in Kuhlmann et al. (2020). [VU]
11. ⸗ Colletes fodiens (Fourcroy, 1785) in Kuhlmann et al. (2020); Markov (2017); Petrik (1958); Rafajlović and Seleši (1958). [VU]
12. ▪ Colletes foveolaris Pérez, 1903 in Kuhlmann et al. (2020). [LC]
13. Colletes gallicus Radoszkowski, 1891 in Rafajlović and Seleši (1958). [LC]
14. ▪ Colletes graeffei Alfken, 1900 in Kuhlmann et al. (2020). [EN]
15. Colletes hederae Schmidt & Westrich, 1993 in Kuhlmann et al. (2020); Burger (2010). [LC]
16. Colletes hylaeiformis Eversmann, 1852 in Kuhlmann et al. (2020); Rafajlović and Seleši (1958); Vorgin (1918); Mocsáry (1897). [LC]
17. ▪ Colletes inexpectatus Noskiewicz, 1936 in Kuhlmann et al. (2020). [LC]
18. ⸗ Colletes maidli Noskiewicz, 1936 in Kuhlmann et al. (2020); Markov (2017). [LC]
19. Colletes marginatus Smith, 1846 in Kuhlmann et al. (2020); Petrik (1958); Rafajlović and Seleši (1958); as Colletes marginata in Apfelbeck (1896). [LC]
20. ▪ Colletes meyeri Noskiewicz, 1936 in Kuhlmann et al. (2020). [EN]
21. ▪ Colletes mlokossewiczi Radoszkowski, 1891 in Kuhlmann et al. (2020). [LC]
22. ⸗ Colletes nasutus Smith, 1853 in Kuhlmann et al. (2020); Mudri-Stojnić (2018); Markov (2017); Rafajlović and Seleši (1958); Živojinović (1950); Lebedev (1931); Mocsáry (1897). [EN]
23. ▪ Colletes nigricans Gistel, 1857 in Kuhlmann et al. (2020). [LC]
24. Colletes punctatus Mocsáry, 1877 in Rafajlović and Seleši (1958); Vorgin (1918); Mocsáry (1897). [EN]
25. ▪ Colletes senilis (Eversmann, 1852) in Kuhlmann et al. (2020). [DD]
26. Colletes succinctus (Linnaeus, 1785) in Kuhlmann et al. (2020); Petrik (1958); Rafajlović and Seleši (1958); Živojinović (1950); Lebedev (1931); Vorgin (1918). [NT]
27. ⸗ Colletes similis Schenck, 1853 in Kuhlmann et al. (2020); Mudri-Stojnić (2018); Petrik (1958); Rafajlović and Seleši (1958); Živojinović (1950); Lebedev (1931); as Colletes picistigma Thomson, 1872 in Vorgin (1918); Mocsáry (1897). [LC]

*Hylaeus* Fabricius, 1793 (42 species)

1. ▪ Hylaeus adriaticus (Warncke, 1972) in Kuhlmann et al. (2020). [DD]
2. ▪ Hylaeus alpinus (Morawitz, 1867) in Kuhlmann et al. (2020). [DD]
3. Hylaeus angustatus (Schenck, 1861) in Kuhlmann et al. (2020); as Prosopis angustata Schenck, 1861 in Rafajlović and Seleši (1958); Živojinović (1950). [LC]
4. ⸗ Hylaeus annularis (Kirby, 1802) in Markov (2017); Markov et al. (2016); AĐ coll.; as Prosopis annularis (Kirby, 1802) in Rafajlović and Seleši (1958); Živojinović (1950). Note: It is difficult to be certain about the correct status of specimens in the literature before Notton and Dathe (2008) who pointed out the confusion regarding previously understood interpretation of the name. [DD]
5. Hylaeus annulatus (Linnaeus, 1758) in Kuhlmann et al. (2020); as Prosopis annulata (Linnaeus, 1758) in Vorgin (1955); Lebedev (1931). [DD]
6. ⸗ Hylaeus brevicornis Nylander, 1852 in Kuhlmann et al. (2020); Mudri-Stojnić (2018); Markov (2017); Markov et al. (2016); as Prosopis brevicornis (Nylander, 1852) in Rafajlović and Seleši (1958); Lebedev (1931); Apfelbeck (1896). [LC]
7. ▪ Hylaeus clypearis (Schenck, 1853) in Kuhlmann et al. (2020). [LC]
8. ⸗ Hylaeus communis Nylander, 1852 in Kuhlmann et al. (2020); Mudri-Stojnić (2018); AĐ coll.; as Prosopis communis (Nylander, 1852) in Rafajlović and Seleši (1958); Lebedev (1931); Apfelbeck (1896). [LC]
9. ⸗ Hylaeus confusus Nylander, 1852 in Kuhlmann et al. (2020); AĐ coll.; as Prosopis confusa (Nylander, 1852) in Rafajlović and Seleši (1958); Apfelbeck (1896). [LC]
10. ▪ Hylaeus coriaceus (Pérez, 1895) in Kuhlmann et al. (2020). [DD]
11. ⸗ Hylaeus cornutus Curtis, 1831 in Mudri-Stojnić (2018); as Prosopis cornuta (Curtis, 1831) in Rafajlović and Seleši (1958); Vorgin (1955, 1918); Mocsáry (1897). [LC]
12. ▪ Hylaeus crassanus (Warncke, 1972) in Kuhlmann et al. (2020). [NT]
13. Hylaeus difformis (Eversmann, 1852) in Kuhlmann et al. (2020); as Prosopis difformis Eversmann, 1852 in Rafajlović and Seleši (1958); Živojinović (1950); Vorgin (1918); Mocsáry (1897). [LC]
14. Hylaeus dilatatus (Kirby, 1802) in Kuhlmann et al. (2020); as Prosopis dilatata (Kirby, 1802) in Apfelbeck (1896). Note: It is difficult to be certain about the correct status of specimens in the literature before Notton and Dathe (2008) who pointed out the confusion regarding previously understood interpretation of the name. [LC]
15. Hylaeus duckei (Alfken, 1904) in Kuhlmann et al. (2020); as Prosopis duckei Alfken, 1904 in Rafajlović and Seleši (1958). [DD]
16. Hylaeus euryscapus Förster, 1871 in Kuhlmann et al. (2020); as Prosopis euryscapus (Föerster, 1871) in Rafajlović and Seleši (1958); Prosopis euryscapa in Vorgin (1955, 1918); Mocsáry (1897). Note: It is difficult to be certain about the correct status of specimens in the literature before Notton and Dathe (2008) who pointed out the confusion regarding previously understood interpretation of the name H. annularis. [DD]
17. Hylaeus gibbus Saunders, 1850 in Kuhlmann et al. (2020); as Prosopis gibba (Saunders, 1850) in Rafajlović and Seleši (1958); Lebedev (1931). [LC]
18. ▪ Hylaeus gredleri Förster, 1871 in Kuhlmann et al. (2020). [LC]
19. Hylaeus hyalinatus Smith, 1842 in Kuhlmann et al. (2020); as Prosopis hyalinata Smith, 1842 in Rafajlović and Seleši (1958); Mocsáry (1897); Apfelbeck (1896). [LC]
20. ▪ Hylaeus hyperpunctatus (Strand, 1909) in Kuhlmann et al. (2020). [DD]
21. ▪ Hylaeus imparilis Förster, 1871 in Kuhlmann et al. (2020). [LC]
22. Hylaeus incongruus Förster, 1871 as Prosopis genalis Thoms. in Vorgin (1918). [DD]
23. ▪ Hylaeus kahri Förster, 1871 in Kuhlmann et al. (2020). [DD]
24. Hylaeus leptocephalus (Morawitz, 1870) in Kuhlmann et al. (2020); as Prosopis bisinuata Förster, 1871 in Rafajlović and Seleši (1958). [LC]
25. ⸗ Hylaeus lineolatus (Schenck, 1861) in Kuhlmann et al. (2020); AĐ coll.; ZM coll.; as Prosopis lineolata Schenck, 1861 in Rafajlović and Seleši (1958); Lebedev (1931). [LC]
26. Hylaeus meridionalis Förster, 1871 in Kuhlmann et al. (2020); as Prosopis meridionalis Förster, 1871 in Rafajlović and Seleši (1958); Mocsáry (1897). [DD]
27. ⸗ Hylaeus nigritus (Fabricius, 1798) in Kuhlmann et al. (2020); AĐ coll.; as Prosopis nigrita (Fabricius, 1798) in Lebedev (1931). [LC]
28. ▪ Hylaeus nivaliformis Dathe, 1977 in Kuhlmann et al. (2020). [DD]
29. ▪ Hylaeus pfankuchi (Alfken, 1919) in Kuhlmann et al. (2020). [LC]
30. Hylaeus pictipes Nylander, 1852 in Kuhlmann et al. (2020); as Prosopis pictipes (Nylander, 1852) in Rafajlović and Seleši (1958); Vorgin (1918). [LC]
31. Hylaeus punctatus (Brullé, 1832) in Kuhlmann et al. (2020); as Prosopis punctata Brullé, 1832 in Rafajlović and Seleši (1958). [LC]
32. ▪ Hylaeus punctulatissimus Smith, 1842 in Kuhlmann et al. (2020). [DD]
33. ▪ Hylaeus punctus Förster, 1871 in Kuhlmann et al. (2020). [DD]
34. ▪ Hylaeus rugicollis Morawitz, 1873 in Kuhlmann et al. (2020). [DD]
35. ▪ Hylaeus scutellatus (Spinola, 1838) in Kuhlmann et al. (2020). [DD]
36. Hylaeus signatus (Panzer, 1798) in Kuhlmann et al. (2020); as Prosopis pratensis (Geoffroy in Fourcroy, 1785) in Rafajlović and Seleši (1958); Vorgin (1955); as Prosopis bipunctata (Fabricius, 1798) in Mocsáry (1897); Apfelbeck (1896). [LC]
37. Hylaeus sinuatus (Schenck, 1853) in Kuhlmann et al. (2020); Petrik (1958); as Prosopis minuta (Fabricius, 1793) in Rafajlović and Seleši (1958); as Prosopis sinuata (Schenck, 1853) in Mocsáry (1897). [LC]
38. ▪ Hylaeus soror (Pérez, 1903) in Kuhlmann et al. (2020). [DD]
39. Hylaeus styriacus Förster, 1871 in Kuhlmann et al. (2020); as Prosopis styriaca (Förster, 1871) in Rafajlović and Seleši (1958). [DD]
40. ▪ Hylaeus taeniolatus Förster, 1871 in Kuhlmann et al. (2020). [LC]
41. ▪ Hylaeus tyrolensis Förster, 1871 in Kuhlmann et al. (2020). [DD]
42. Hylaeus variegatus (Fabricius, 1798) in Kuhlmann et al. (2020); as Prosopis variegata (Fabricius, 1798) in Rafajlović and Seleši (1958); Lebedev (1931); Mocsáry (1897); Apfelbeck (1896). [LC]

### Halictidae (12 genera; 138 species)

*Ceylalictus* Strand, 1913 (1 species)

1. Ceylalictus variegatus (Olivier, 1789) in Kuhlmann et al. (2020); as Nomioides jucunda Morawitz, 1874 in Petrik (1958); as Nomioides variegatus (Olivier, 1789) in Rafajlović and Seleši (1958); Mocsáry (1897). [LC]

*Dufourea* Lepeletier, 1841 (4 species)

* Dufourea alpina Morawitz, 1865 in Apfelbeck (1896). [LC]
2. Dufourea dentiventris (Nylander, 1848) in Kuhlmann et al. (2020); as Halictoides dentiventris Nylander, 1848 in Lebedev (1931). [NT]
3. ⸗ Dufourea inermis (Nylander, 1848) in Kuhlmann et al. (2020); AĐ coll. [NT]
4. ▪ Dufourea minuta Lepeletier, 1841 in Kuhlmann et al. (2020). [NT]

*Halictus* Latreille, 1804 (18 species)

1. ⸗ Halictus asperulus Pérez, 1895 in Kuhlmann et al. (2020); Markov (2017); Markov et al. (2016); Grozdanić (1972a); Rafajlović and Seleši (1958); Lebedev (1931). [DD]
2. ⸗ Halictus brunnescens (Eversmann, 1852) in Markov (2017); Markov et al. (2016). [DD]
3. ▪ Halictus carinthiacus Blüthgen, 1936 in Kuhlmann et al. (2020). [EN]
4. Halictus cochlearitarsis Dours, 1872 in Grozdanić (1966); Rafajlović and Seleši (1958). [LC]
5. ⸗ Halictus compressus (Walkenaer, 1802) as Halictus eurygnathus Blüthgen, 1930 in Markov (2017); Markov et al. (2016); Živojinović (1950). [LC]
6. ⸗ Halictus fulvipes (Klug, 1817) in Mudri-Stojnić (2018); Markov (2017); Markov et al. (2016); Mučalica (1968); Lebedev (1931). [LC]
7. ⸗ Halictus langobardicus Blüthgen, 1944 in Kuhlmann et al. (2020); Mudri-Stojnić (2018); Rafajlović and Seleši (1958). [LC]
8. ⸗ Halictus maculatus Smith, 1848 in Kuhlmann et al. (2020); Mudri-Stojnić (2018); Markov (2017); Markov et al. (2016); Mudri-Stojnić et al. (2012); Grozdanić (1972a); Petrik (1958); Rafajlović and Seleši (1958); Vorgin (1955); Živojinović (1950); Lebedev (1931); Mocsáry (1897); Apfelbeck (1896); Korlević (1890); AĐ coll.; ZM coll. [LC]
9. Halictus mucoreus Eversmann, 1852 in Vorgin (1955, 1918); Mocsáry (1897). [DD]
10. ⸗ Halictus patellatus Morawitz, 1873 in Mudri-Stojnić (2018); Markov (2017); Markov et al. (2016); Mudri-Stojnić et al. (2012); Rafajlović and Seleši (1958); Vorgin (1955); Mocsáry (1897); Apfelbeck (1896); AZ coll.; ZM coll. [LC]
11. ⸗ Halictus quadricinctus (Fabricius, 1776) in Mudri-Stojnić (2018); Markov (2017); Markov et al. (2016); Mudri-Stojnić et al. (2012); Grozdanić (1971b, 1966); Vasić (1967); Grozdanić and Vasić (1966a, 1965c); Rafajlović and Seleši (1958); Vorgin (1955); Anđelković (1949); Lebedev (1931); Mocsáry (1897); Apfelbeck (1896); as Halictus quadristrigatus Latreille, 1805 in Petrik (1958); Vorgin (1955); Korlević (1890). [NT]
12. Halictus resurgens Nurse, 1903 as Halictus holtzi Schulz, 1906 in Lebedev (1931). [LC]
13. ⸗ Halictus rubicundus (Christ, 1791) in Markov (2017); Markov et al. (2016); Rafajlović and Seleši (1958); Vorgin (1955); Živojinović (1950); Lebedev (1931); AĐ coll.; as Halictus quadrifasciatus Smith, 1870 in Mudri-Stojnić (2018). [LC]
14. ⸗ Halictus sajoi Blüthgen, 1923 in Grozdanić (1971a); ZM coll.; as Halictus veneticus Móczár, 1967 in Rafajlović and Seleši (1958). [DD]
15. ⸗ Halictus scabiosae (Rossi, 1790) in Vasić (1979a); Grozdanić (1971b, 1970, 1966, 1960); Grozdanić and Vasić (1966a, 1965c); Krunić (1959); Vorgin (1955); Petrik (1958); Rafajlović and Seleši (1958); Lebedev (1931); Mocsáry (1897); Apfelbeck (1896); ZM coll.; FSUNS. [LC]
16. ⸗ Halictus sexcinctus (Fabricius, 1775) in Mudri-Stojnić (2018); Markov (2017); Markov et al. (2016); Grozdanić (1960, 1950a); Petrik (1958); Rafajlović and Seleši (1958); Živojinović (1950); Lebedev (1931); Apfelbeck (1896); Mocsáry (1897); Korlević (1890); AZ coll.; ZM coll. [LC]
17. ⸗ Halictus simplex Blüthgen, 1923 in Kuhlmann et al. (2020); Mudri-Stojnić (2018); Mudri-Stojnić et al. (2012); Rafajlović and Seleši (1958); Živojinović (1950); AĐ coll.; AZ coll.; ZM coll. [LC]
18. ⸗ Halictus tetrazonius (Klug, 1817) in Petrik (1958); Vorgin (1955); Mocsáry (1897); Apfelbeck (1896); SG coll.; ZM coll. [DD]

*Lasioglossum* Curtis, 1833 (72 species)

1. Lasioglossum aeratum (Kirby, 1802) as Halictus viridiaeneus Blüthgen, 1918 in Rafajlović and Seleši (1958); Vorgin (1955); as Halictus aeratus (Kirby, 1802) in Mocsáry (1897). [LC]
2. ⸗ Lasioglossum albipes (Fabricius, 1781) in Mudri-Stojnić (2018); Mudri-Stojnić et al. (2012); as Halictus albipes (Fabricius, 1781) in Rafajlović and Seleši (1958); Živojinović (1950); Lebedev (1931); Mocsáry (1897); as Halictus obovatus Kirby in Petrik (1958). [LC]
3. ▪ Lasioglossum alpigenum (Dalla Torre, 1877) in Kuhlmann et al. (2020). [LC]
4. ▪ Lasioglossum angusticeps (Perkins, 1895) in Kuhlmann et al. (2020). [NT]
5. ▪ Lasioglossum apostoli Ebmer, 1970 in Kuhlmann et al. (2020). [DD]
6. Lasioglossum bischoffi (Blüthgen, 1931) in Kuhlmann et al. (2020); as Halictus bischoffi Blüthgen, 1931 in Rafajlović and Seleši (1958). [DD]
7. ⸗ Lasioglossum brevicorne (Schenck, 1868) in Markov (2017); Markov et al. (2016); AĐ coll.; ZM coll.; as Halictus brevicornis Schenck, 1870 [“1869”] in Rafajlović and Seleši (1958); Vorgin (1955). [NT]
8. ⸗ Lasioglossum calceatum (Scopoli, 1763) in Kuhlmann et al. (2020); Mudri-Stojnić (2018); Markov (2017); Markov et al. (2016); AĐ coll.; as Halictus calceatus (Scopoli, 1763) in Rafajlović and Seleši (1958); Živojinović (1950); Lebedev (1931); Mocsáry (1897); Apfelbeck (1896); as Halictus cylindricus (Fabricius, 1793) in Petrik (1958); Korlević (1890). [LC]
9. ⸗ Lasioglossum clypeare (Schenck, 1853) in Markov (2017); Markov et al. (2016); as Halictus clypearis (Schenck, 1853) in Vorgin (1955); Živojinović (1950); Lebedev (1931). [NT]
10. Lasioglossum convexiusculum (Schenck, 1853) as Halictus convexiusculus (Schenck, 1853) in Rafajlović and Seleši (1958). [NT]
11. Lasioglossum corvinum (Morawitz, 1877) in Kuhlmann et al. (2020); as Halictus corvinus Morawitz, 1877 in Rafajlović and Seleši (1958); Lebedev (1931). [LC]
12. Lasioglossum costulatum (Kriechbaumer, 1873) as Halictus costulatus Kriechbaumer, 1873 in Rafajlović and Seleši (1958); Živojinović (1950); Lebedev (1931); Mocsáry (1897). [NT]
13. ⸗ Lasioglossum crassepunctatum (Blüthgen, 1923) in Kuhlmann et al. (2020); Mudri-Stojnić (2018); as Halictus crassepunctatus Blüthgen, 1923 in Rafajlović and Seleši (1958); Vorgin (1955). [DD]
14. ⸗ Lasioglossum discum (Smith, 1853) in Kuhlmann et al. (2020); Mudri-Stojnić (2018); Markov (2017); Markov et al. (2016); Mudri-Stojnić et al. (2012); ZM coll.; as Halictus morbillosus Kriechbaumer, 1873 in Grozdanić (1971b); Rafajlović and Seleši (1958); Vorgin (1955); Živojinović (1950); Lebedev (1931); Mocsáry (1897); Apfelbeck (1896). [LC]
15. Lasioglossum elegans (Lepeletier, 1841) in Kuhlmann et al. (2020); as Halictus elegans Lepeletier, 1841 in Rafajlović and Seleši (1958); Mocsáry (1897). [DD]
16. ⸗ Lasioglossum euboeense (Strand, 1909) as Halictus euboeensis Strand, 1909 in Rafajlović and Seleši (1958); Vorgin (1955); SG coll. [DD]
17. ⸗ Lasioglossum fratellum (Pérez, 1903) in Kuhlmann et al. (2020); AĐ coll.; ZM coll. [LC]
18. ⸗ Lasioglossum fulvicorne (Kirby, 1802) in AĐ coll.; as Halictus fulvicornis (Kirby, 1802) in Rafajlović and Seleši (1958); Živojinović (1950). [LC]
19. ⸗ Lasioglossum glabriusculum (Morawitz, 1872) in Mudri-Stojnić (2018); Markov (2017); Markov et al. (2016); Mudri-Stojnić et al. (2012); as Halictus glabriusculus Morawitz, 1872 in Rafajlović and Seleši (1958); Vorgin (1955); Živojinović (1950); Lebedev (1931). [LC]
20. Lasioglossum griseolum (Morawitz, 1872) as Halictus griseolus Morawitz, 1872 in Rafajlović and Seleši (1958); Vorgin (1955). [LC]
21. ▪ Lasioglossum intermedium (Schenck, 1868) in Kuhlmann et al. (2020). [NT]
22. ⸗ Lasioglossum interruptum (Panzer, 1798) in Markov (2017); SG coll.; as Halictus interruptus (Panzer, 1798) in Grozdanić and Mučalica (1968b); Rafajlović and Seleši (1958); Vorgin (1955); Mocsáry (1897); Apfelbeck (1896). [LC]
23. ▪ Lasioglossum kussariense (Blüthgen, 1925) in Kuhlmann et al. (2020). [DD]
24. Lasioglossum laeve (Kirby, 1802) in Kuhlmann et al. (2020); as Halictus laevis (Kirby, 1802) in Vorgin (1918). [EN]
25. Lasioglossum laevigatum (Kirby, 1802) in Kuhlmann et al. (2020); as Halictus laevigatus (Kirby, 1802) in Rafajlović and Seleši (1958); Živojinović (1950). [NT]
26. ⸗ Lasioglossum laticeps (Schenck, 1868) in Kuhlmann et al. (2020); Mudri-Stojnić (2018); Markov (2017); as Halictus laticeps Schenck, 1868 in Rafajlović and Seleši (1958); Živojinović (1950); Lebedev (1931). [LC]
27. ⸗ Lasioglossum lativentre (Schenck, 1853) in Kuhlmann et al. (2020); Mudri-Stojnić (2018); Mudri-Stojnić et al. (2012); ZM coll.; as Halictus lativentris Schenck, 1853 in Rafajlović and Seleši (1958); Živojinović (1950); Lebedev (1931). [LC]
28. Lasioglossum leucopus (Kirby, 1802) in Kuhlmann et al. (2020); as Halictus leucopus (Kirby, 1802) in Rafajlović and Seleši (1958); Živojinović (1950). [LC]
29. ⸗ Lasioglossum leucozonium (Schrank, 1781) in Mudri-Stojnić (2018); Markov (2017); Mudri-Stojnić et al. (2012); AĐ coll.; ZM coll.; as Halictus leucozonius (Schrank, 1781) in Grozdanić (1971b); Rafajlović and Seleši (1958); Vorgin (1955, 1918); Živojinović (1950); Lebedev (1931); Mocsáry (1897); Apfelbeck (1896). [LC]
30. Lasioglossum limbellum (Morawitz, 1876) in Kuhlmann et al. (2020); as Halictus limbellus Morawitz, 1876 in Rafajlović and Seleši (1958). [DD]
31. ⸗ Lasioglossum lineare (Schenck, 1868) in Mudri-Stojnić (2018); Markov (2017); Markov et al. (2016); SG coll.; as Halictus linearis Schenck, 1868 in Grozdanić (1971b); Grozdanić and Vasić (1966a); Rafajlović and Seleši (1958); Vorgin (1955); Živojinović (1950); Lebedev (1931). [DD]
32. ▪ Lasioglossum lissonotum (Noskiewicz, 1926) in Kuhlmann et al. (2020). [DD]
33. Lasioglossum lucidulum (Schenck, 1861) in Kuhlmann et al. (2020); as Halictus lucidulus (Schenck, 1861) in Rafajlović and Seleši (1958); Vorgin (1955). [LC]
34. ⸗ Lasioglossum majus (Nylander, 1852) in Kuhlmann et al. (2020); Markov (2017); Markov et al. (2016); AĐ coll.; as Halictus major Nylander, 1852 in Rafajlović and Seleši (1958); Živojinović (1950); Lebedev (1931); Vorgin (1918). [NT]
35. Lasioglossum mandibulare (Morawitz, 1866) in Kuhlmann et al. (2020); as Halictus mandibularis Morawitz, 1866 in Rafajlović and Seleši (1958); Vorgin (1955). [NT]
36. ⸗ Lasioglossum malachurum (Kirby, 1802) in Mudri-Stojnić (2018); Markov (2017); Markov et al. (2016); Mudri-Stojnić et al. (2012); SG coll.; as Halictus malachurus (Kirby, 1802) in Grozdanić (1971b, 1969c, 1966, 1960); Grozdanić and Vasić (1970, 1965c); Krunić (1959); Rafajlović and Seleši (1958); Vorgin (1955); Živojinović (1950); Lebedev (1931). [LC]
37. ⸗ Lasioglossum marginatum (Brullé, 1832) in Kuhlmann et al. (2020); Mudri-Stojnić (2018); Markov (2017); Markov et al. (2016); ZM coll.; as Halictus marginatus Brullé, 1832 in Vasić (1979b, 1970, 1966); Grozdanić (1971b, 1970, 1969c, 1966, 1960, 1956); Grozdanić and Vasić (1965c); Krunić (1959); Rafajlović and Seleši (1958); Vorgin (1955); Lebedev (1931); as Halictus fasciatellus Schenck, 1868 in Vorgin (1955); Mocsáry (1897); Apfelbeck (1896). [LC]
38. ▪ Lasioglossum marginellum (Schenck, 1853) in Kuhlmann et al. (2020). [NT]
39. Lasioglossum mesosclerum (Pérez, 1903) in Kuhlmann et al. (2020); as Halictus mesosclerus Pérez, 1903 in Rafajlović and Seleši (1958); Vorgin (1955). [DD]
40. Lasioglossum minutissimum (Kirby, 1802) in Kuhlmann et al. (2020); as Halictus minutissimus (Kirby, 1802) in Rafajlović and Seleši (1958). [LC]
41. Lasioglossum minutulum (Schenck, 1853) as Halictus minutulus (Schenck, 1853) in Rafajlović and Seleši (1958); Živojinović (1950). [NT]
42. ⸗ Lasioglossum morio (Fabricius, 1793) in Kuhlmann et al. (2020); AĐ coll.; as Halictus morio (Fabricius, 1793) in Rafajlović and Seleši (1958); Vorgin (1955); Živojinović (1950); Lebedev (1931); Mocsáry (1897); Apfelbeck (1896). [LC]
43. ⸗ Lasioglossum nigripes (Lepeletier, 1841) in Kuhlmann et al. (2020); Mudri-Stojnić (2018); Markov (2017); Markov et al. (2016); ZM coll.; as Halictus nigripes Lepeletier, 1841 in Vasić (1979b); Grozdanić and Vasić (1966a); Vorgin (1955); Živojinović (1950); Lebedev (1931); as Halictus vulpinus Nylander, 1853 (nec Fabricius, 1804) in Rafajlović and Seleši (1958); Mocsáry (1897). [LC]
44. ⸗ Lasioglossum nitidiusculum (Kirby, 1802) in Kuhlmann et al. (2020); AĐ coll.; as Halictus nitidiusculus (Kirby, 1802) in Rafajlović and Seleši (1958); Lebedev (1931). [LC]
45. Lasioglossum nitidulum (Fabricius, 1804) as Halictus aeneidorsum Alfken, 1921 in Rafajlović and Seleši (1958); Vorgin (1955). [LC]
46. ⸗ Lasioglossum obscuratum (Morawitz, 1876) in Kuhlmann et al. (2020); ZM coll.; as Halictus obscuratus Morawitz, 1876 in Rafajlović and Seleši (1958); Živojinović (1950); Mocsáry (1897). [DD]
47. ⸗ Lasioglossum pallens (Brullé, 1832) in Kuhlmann et al. (2020); Markov (2017); Markov et al. (2016); as Halictus lineolatus Lepeletier, 1841 in Mocsáry (1897). [LC]
48. Lasioglossum parvulum (Schenck, 1853) in Kuhlmann et al. (2020); as Halictus minutus (Kirby, 1802) in Rafajlović and Seleši (1958); Živojinović (1950). [LC]
49. ⸗ Lasioglossum pauxillum (Schenck, 1853) in Mudri-Stojnić (2018); Markov (2017); Markov et al. (2016); Mudri-Stojnić et al. (2012); AĐ coll.; as Halictus pauxillus (Schenck, 1853) in Rafajlović and Seleši (1958); Živojinović (1950); Lebedev (1931). [LC]
50. ⸗ Lasioglossum politum (Schenck, 1853) in Markov (2017); Markov et al. (2016); as Halictus politus (Schenck, 1853) in Rafajlović and Seleši (1958); Vorgin (1955, 1918); Živojinović (1950); Lebedev (1931). [LC]
51. ▪ Lasioglossum pseudocaspicum (Blüthgen, 1923) in Kuhlmann et al. (2020). [DD]
52. Lasioglossum punctatissimum (Schenck, 1853) in Kuhlmann et al. (2020); as Halictus punctatissimus (Schenck, 1859) in Lebedev (1931). [LC]
53. ⸗ Lasioglossum puncticolle (Morawitz, 1872) in Kuhlmann et al. (2020); Mudri-Stojnić (2018); Markov (2017); Markov et al. (2016); as Halictus puncticollis Morawitz, 1872 in Rafajlović and Seleši (1958); Lebedev (1931). [LC]
54. ⸗ Lasioglossum pygmaeum (Schenck, 1853) in Markov (2017); Markov et al. (2016); ZM coll.; as Halictus pygmaeus (Fabricius, 1804) in Rafajlović and Seleši (1958); Vorgin (1955). [NT]
55. Lasioglossum quadrinotatulum (Schenck, 1861) in Kuhlmann et al. (2020); as Halictus quadrinotatulus (Schenck, 1859) in Rafajlović and Seleši (1958); Vorgin (1955). [NT]
56. Lasioglossum quadrinotatum (Kirby, 1802) in Kuhlmann et al. (2020); as Halictus quadrinotatus (Kirby, 1802) in Vorgin (1955); Apfelbeck (1896). [NT]
57. ▪ Lasioglossum rufitarse (Zetterstedt, 1838) in Kuhlmann et al. (2020). [LC]
58. Lasioglossum semilucens (Alfken, 1914) in Kuhlmann et al. (2020); as Halictus semilucens Alfken, 1914 in Rafajlović and Seleši (1958). [LC]
59. Lasioglossum setulellum (Strand, 1909) as Halictus setulellus Strand, 1909 in Vorgin (1955). [NT]
60. ▪ Lasioglossum setulosum (Strand, 1909) in Kuhlmann et al. (2020). [NT]
61. ⸗ Lasioglossum sexnotatum (Kirby, 1802) in Kuhlmann et al. (2020); AĐ coll.; as Halictus sexnotatus (Kirby, 1802) in Vorgin (1918); Mocsáry (1897). [NT]
62. ⸗ Lasioglossum sexstrigatum (Schenck, 1868) in ZM coll.; as Halictus sexstrigatus Schenck, 1870[“1869”] in Rafajlović and Seleši (1958); Vorgin (1955). [LC]
63. Lasioglossum smeathmanellum (Kirby, 1802) as Halictus smeathmanellus K. in Rafajlović and Seleši (1958); Vorgin (1955, 1918); Živojinović (1950); Apfelbeck (1896). [LC]
64. Lasioglossum subfasciatum (Imhoff, 1832) as Halictus subfasciatus (Imhoff, 1832) in Lebedev (1931). [EN]
65. ▪ Lasioglossum subfulvicorne (Blüthgen, 1934) in Kuhlmann et al. (2020). [LC]
66. Lasioglossum tarsatum (Schenck, 1868) as Halictus tarsatus Schenck, 1868 in Rafajlović and Seleši (1958); Vorgin (1955). [NT]
67. ⸗ Lasioglossum trichopygum (Blüthgen, 1923) in ZM coll.; as Halictus trichopygus Blüthgen, 1923 in Rafajlović and Seleši (1958); Grozdanić (1966); Grozdanić and Vasić (1966a). [DD]
68. Lasioglossum tricinctum (Schenck, 1874) in Kuhlmann et al. (2020); as Halictus tricinctus Schenck, 1874 in Živojinović (1950). [DD]
69. ⸗ Lasioglossum truncaticolle (Morawitz, 1877[“1878”]) in Mudri-Stojnić (2018); Markov (2017); Markov et al. (2016); as Halictus truncaticollis Morawitz, 1877[“1878”] in Vorgin (1955); Lebedev (1931). [DD]
70. ⸗ Lasioglossum villosulum (Kirby, 1802) in Kuhlmann et al. (2020); Mudri-Stojnić (2018); Markov (2017); Markov et al. (2016); SG coll.; as Halictus villosulus Kirby, 1802 in Rafajlović and Seleši (1958); Vorgin (1955); Lebedev (1931); Apfelbeck (1896). [LC]
71. ⸗ Lasioglossum xanthopus (Kirby, 1802) in Kuhlmann et al. (2020); Mudri-Stojnić (2018); as Halictus xanthopus (Kirby, 1802) in Petrik (1958); Rafajlović and Seleši (1958); Vorgin (1955, 1918); Lebedev (1931); Mocsáry (1897); as Lasioglossum xanthopum (Kirby, 1802) in ZM coll. [NT]
72. Lasioglossum zonulum (Smith, 1848) as Halictus zonulus Smith, 1848 in Petrik (1958); Rafajlović and Seleši (1958); Vorgin (1955, 1918); Lebedev (1931); Mocsáry (1897); Apfelbeck (1896). [LC]

*Nomiapis* Cockerell, 1919 (3 species)

1. ⸗ Nomiapis bispinosa (Brullé, 1832) as Nomia ruficornis Spinola, 1839 in Grozdanić (1971b); Petrik (1958); Rafajlović and Seleši (1958); Lebedev (1931); Mocsáry (1897); as Nomia unidentata Oliver, 1812 in Mudri-Stojnić (2018). [LC]
2. ⸗ Nomiapis diversipes (Latreille, 1806) in Mudri-Stojnić (2018); ZM coll.; as Nomia diversipes Latreille, 1806 in Markov (2017); Mudri-Stojnić et al. (2012); Rafajlović and Seleši (1958); Lebedev (1931); Mocsáry (1897); Apfelbeck (1896); AZ coll.; as Pseudapis diversipes (Latreille, 1806) in Markov et al. (2016). [LC]
3. ⸗ Nomiapis femoralis (Pallas, 1773) in Kuhlmann et al. (2020); ZM coll.; as Nomia femoralis (Pallas, 1773) in Petrik (1958); Rafajlović and Seleši (1958); Mocsáry (1897). [DD]

*Nomioides* Schenck, 1867 (1 species)

1. Nomioides minutissimus (Rossi, 1790) in Kuhlmann et al. (2020); Rafajlović and Seleši (1958); as Nomioides pulchellus Schenck, 1859 in Mocsáry (1897). [LC]

*Rhophitoides* Schenck, 1861 (1 species)

1. ⸗ Rhophitoides canus (Eversmann, 1852) in Kuhlmann et al. (2020); Mudri-Stojnić (2018); Mudri-Stojnić et al. (2012); as Rophites canus Eversmann, 1852 in Rafajlović and Seleši (1958); Vorgin (1918); Mocsáry (1897); Apfelbeck (1896); ZM coll. [LC]

*Rophites* Spinola, 1808 (2 species)

1. ⸗ Rophites hartmanni Friese, 1902 in Markov (2017); Markov et al. (2016); Rafajlović and Seleši (1958); Vorgin (1918); AZ coll. [DD]
2. ⸗ Rophites quinquespinosus Spinola, 1808 in Mudri-Stojnić (2018); Mudri-Stojnić et al. (2012); Grozdanić (1971b); Rafajlović and Seleši (1958); Lebedev (1931); Apfelbeck (1896); Mocsáry (1897); ZM coll. [NT]

*Seladonia* Robertson, 1918 (8 species)

1. Seladonia confusa (Smith, 1853) as Halictus perkinsi Blüthgen, 1926 in Rafajlović and Seleši (1958); Vorgin (1955); Živojinović (1950). [LC]
2. ▪ Seladonia gavarnica (Pérez, 1903) as Halictus gavarnicus Pérez, 1903 in Kuhlmann et al. (2020). [LC]
3. ⸗ Seladonia kessleri (Bramson, 1879) as Halictus kessleri Bramson, 1879 in Mudri-Stojnić (2018); Markov (2017); Markov et al. (2016); Mudri-Stojnić et al. (2012); Vasić (1979b); Grozdanić (1974, 1972b, 1971b, 1966); Grozdanić and Vasić (1966a); Rafajlović and Seleši (1958); Lebedev (1931); ZM coll. [LC]
4. ⸗ Seladonia seladonia (Fabricius, 1794) as Halictus seladonius (Fabricius, 1794) in Markov (2017); Markov et al. (2016); as Halictus geminatus Pérez, 1903 in Rafajlović and Seleši (1958); Vorgin (1955); Živojinović (1950). [LC]
5. ⸗ Seladonia semitecta (Morawitz, 1873) as Halictus semitectus Morawitz, 1874 in Kuhlmann et al. (2020); Mudri-Stojnić (2018); Mudri-Stojnić et al. (2012); Rafajlović and Seleši (1958); Vorgin (1955); Mocsáry (1897). [EN]
6. ⸗ Seladonia smaragdula (Vachal, 1895) as Halictus smaragdulus Vachal, 1895 in Markov (2017); Markov et al. (2016); Rafajlović and Seleši (1958); Vorgin (1955); AZ coll. [LC]
7. ⸗ Seladonia subaurata (Rossi, 1792) as Halictus subauratus (Rossi, 1792) in Kuhlmann et al. (2020); Mudri-Stojnić (2018); Markov (2017); Markov et al. (2016); Mudri-Stojnić et al. (2012); Rafajlović and Seleši (1958); Živojinović (1950); Lebedev (1931); AZ coll.; as Halictus gramineus Smith, 1849 in Petrik (1958); as Halictus virescens Lepeletier, 1841 in Vorgin (1955); Mocsáry (1897); Apfelbeck (1896). [LC]
8. ⸗ Seladonia tumulorum (Linnaeus, 1758) as Halictus tumulorum (Linnaeus, 1758) in Lebedev (1931); Apfelbeck (1896); AĐ coll.; as Halictus fasciatus Nylander, 1848 in Rafajlović and Seleši (1958); Vorgin (1955); Lebedev (1931). [LC]

*Sphecodes* Latreille, 1802 (25 species)

1. ⸗ Sphecodes albilabris Fabricius, 1793 in Kuhlmann et al. (2020); Markov (2017); Markov et al. (2016); ZM coll.; as Sphecodes fuscipennis (Germar, 1819) in Petrik (1958); Rafajlović and Seleši (1958); Mocsáry (1897); Apfelbeck (1896). [LC]
2. ⸗ Sphecodes alternatus Smith, 1853 in Kuhlmann et al. (2020); Mudri-Stojnić (2018); Mudri-Stojnić et al. (2012). [LC]
3. Sphecodes crassus Thomson, 1870 in Kuhlmann et al. (2020); as Sphecodes divisus Hagens, 1882 in Živojinović (1950); Rafajlović and Seleši (1958). [LC]
4. Sphecodes cristatus Hagens, 1882 in Kuhlmann et al. (2020); Rafajlović and Seleši (1958). Note: Bogusch and Straka (2012) reported this species as absent on Balkan Peninsula. [NT]
5. ▪ Sphecodes croaticus Meyer, 1922 in Kuhlmann et al. (2020). [NT]
6. ⸗ Sphecodes ephippius Linnaeus, 1767 in Kuhlmann et al. (2020); Vorgin (1918); AĐ coll.; ZM coll.; as Sphecodes similis Wesmael, 1836 in Apfelbeck (1896). [LC]
7. Sphecodes ferruginatus Hagens, 1882 in Kuhlmann et al. (2020); Lebedev (1931); as Sphecodes rufescens Hagens, 1874 in Apfelbeck (1896). [LC]
8. Sphecodes geoffrellus Kirby, 1802 in Kuhlmann et al. (2020); as Sphecodes fasciatus Hagens, 1882 in Živojinović (1950). [LC]
9. ⸗ Sphecodes gibbus Linnaeus, 1758 in Kuhlmann et al. (2020); Mudri-Stojnić (2018); Rafajlović and Seleši (1958); Živojinović (1950); Lebedev (1931); Vorgin (1918); Mocsáry (1897); Apfelbeck (1896). [LC]
10. ▪ Sphecodes hyalinatus Hagens, 1882 in Kuhlmann et al. (2020). [NT]
11. ⸗ Sphecodes longulus Hagens, 1882 in Kuhlmann et al. (2020); Markov (2017); Markov et al. (2016); Rafajlović and Seleši (1958). [LC]
12. ▪ Sphecodes majalis Pérez, 1903 in Kuhlmann et al. (2020). [NT]
13. ⸗ Sphecodes miniatus Hagens, 1882 in Kuhlmann et al. (2020); AĐ coll. [LC]
14. ⸗ Sphecodes monilicornis Kirby, 1802 in Kuhlmann et al. (2020); Mudri-Stojnić (2018); Markov (2017); Markov et al. (2016); Grozdanić (1971b); Rafajlović and Seleši (1958); Živojinović (1950); Lebedev (1931); as Sphecodes subquadratus Smith, 1845 in Vorgin (1918); Mocsáry (1897); Apfelbeck (1896). [LC]
15. Sphecodes niger Hagens, 1874 in Kuhlmann et al. (2020); Rafajlović and Seleši (1958). [LC]
16. ⸗ Sphecodes pellucidus Smith, 1845 in Kuhlmann et al. (2020); Markov (2017); Markov et al. (2016); Rafajlović and Seleši (1958); Živojinović (1950); AĐ coll. [LC]
17. ▪ Sphecodes pseudofasciatus Blüthgen, 1925 in Kuhlmann et al. (2020). [DD]
18. Sphecodes puncticeps Thomson, 1870 in Kuhlmann et al. (2020); Rafajlović and Seleši (1958); Živojinović (1950). [LC]
19. Sphecodes reticulatus Thomson, 1870 in Kuhlmann et al. (2020); Rafajlović and Seleši (1958); Lebedev (1931); Mocsáry (1897). [LC]
20. Sphecodes rubicundus Hagens, 1875 in Lebedev (1931). [NT]
21. Sphecodes rufiventris Panzer, 1798 in Kuhlmann et al. (2020); Mocsáry (1897); as Sphecodes subovalis Schenck, 1853 in Rafajlović and Seleši (1958); Lebedev (1931). [LC]
22. Sphecodes scabricollis Wesmael, 1835 in Kuhlmann et al. (2020); Rafajlović and Seleši (1958). [DD]
23. ⸗ Sphecodes schenckii Hagens, 1882 in Grozdanić (1971b); Rafajlović and Seleši (1958); ZM coll. [NT]
24. ▪ Sphecodes spinulosus Hagens, 1875 in Kuhlmann et al. (2020). [NT]
25. ▪ Sphecodes zangherii Noskiewicz, 1931 in Kuhlmann et al. (2020). Note: Bogusch and Straka (2012) stated that the distribution of this species is poorly known due the taxonomical problems in the past, as many specimens of this species (with previously suggested wide distribution in south and central Europe) were missidentified S. croaticus. [DD]

*Systropha* Illiger, 1805 (2 species)

1. ⸗ Systropha curvicornis (Scopoli, 1770) in Mudri-Stojnić (2018); Markov (2017); Markov et al. (2016); Grozdanić (1971b); Grozdanić and Mučalica (1966); Rafajlović and Seleši (1958); Živojinović (1950); Lebedev (1931); Vorgin (1918), Mocsáry (1897); ZM coll. [NT]
2. ⸗ Systropha planidens Giraud, 1861 in Mudri-Stojnić (2018); Markov (2017); Markov et al. (2016); Grozdanić (1971b); Grozdanić and Vasić (1968); Grozdanić and Mučalica (1966); Vorgin (1918); Mocsáry (1897); Apfelbeck (1896); SG coll. [VU]

*Vestitohalictus* Blüthgen, 1961 (2 species)

1. ⸗ Vestitohalictus pollinosus (Sichel, 1860) as Halictus pollinosus Sichel, 1860 in Mudri-Stojnić (2018); Markov (2017); Markov et al. (2016); Mudri-Stojnić et al. (2012); Petrik (1958); Rafajlović and Seleši (1958); as Halictus carinaeventris Fahringer & Friese, 1921 in Mocsáry (1897). [LC]
2. ⸗ Vestitohalictus vestitus (Lepeletier, 1841) as Halictus vestitus Lepeletier, 1841 in Rafajlović and Seleši (1958); Vorgin (1955); ZM coll. [LC]

### Megachilidae (17 genera; 148 species)

*Aglaoapis* Cameron, 1901 (1 species)

1. Aglaoapis tridentata (Nylander, 1848) as Dioxoides tridentata (Nylander, 1848) in Stanisavljević (2000). [LC]

*Anthidiellum* Cockerell, 1904 (1 species)

1. Anthidiellum strigatum Panzer, 1805 in Kuhlmann et al. (2020); Stanisavljević (2000); as Anthidium strigatum (Panzer, 1805) in Rafajlović and Seleši (1958); Vorgin (1955); Mocsáry (1897). [LC]

*Anthidium* Fabricius, 1804 (12 species)

1. Anthidium cingulatum Latreille, 1809 in Kuhlmann et al. (2020); Stanisavljević (2000); Rafajlović and Seleši (1958); Vorgin (1955); Lebedev (1931); Mocsáry (1897). [LC]
2. ▪ Anthidium diadema Latreille, 1809 in Kuhlmann et al. (2020). [DD]
3. ⸗ Anthidium florentinum Fabricius, 1775 in Kuhlmann et al. (2020); Mudri-Stojnić (2018); Stanisavljević (2000); Rafajlović and Seleši (1958); Vorgin (1918); Mocsáry (1897). [LC]
4. Anthidium loti Perris, 1852 in Kuhlmann et al. (2020); Stanisavljević (2000); as Anthidium variegatum (Fabricius, 1781) in Rafajlović and Seleši (1958). [DD]
5. ⸗ Anthidium manicatum Linnaeus, 1758 in Kuhlmann et al. (2020); Mudri-Stojnić (2018); Markov (2017); Markov et al. (2016); Mudri-Stojnić et al. (2012); Stanisavljević (2000); Grozdanić and Vasić (1966a); Petrik (1958); Rafajlović and Seleši (1958); Živojinović (1950); Lebedev (1931); Apfelbeck (1896). [LC]
6. ▪ Anthidium montanum Morawitz, 1864 in Kuhlmann et al. (2020). [NT]
7. ⸗ Anthidium oblongatum Illiger, 1806 in Kuhlmann et al. (2020); Markov (2017); Markov et al. (2016); Krunić et al. (1988); Rafajlović and Seleši (1958); Vorgin (1918); Mocsáry (1897); AĐ coll.; as Proanthidium oblongatum (Illiger, 1806) in Stanisavljević (2000) [LC]
8. ⸗ Anthidium punctatum Latreille, 1809 in Kuhlmann et al. (2020); Markov (2017); Stanisavljević (2000); Rafajlović and Seleši (1958); Živojinović (1950); Mocsáry (1897). [LC]
9. Anthidium septemspinosum Lepeletier, 1841 in Kuhlmann et al. (2020); Stanisavljević (2000); Vorgin (1918). [DD]
10. ▪ Anthidium taeniatum Latreille, 1809 in Kuhlmann et al. (2020). [DD]
11. ▪ Anthidium undulatiforme Friese, 1917 in Kuhlmann et al. (2020). [NT]
12. ▪ Anthidium undulatum Dours, 1873 in Kuhlmann et al. (2020). [LC]

*Chelostoma* Latreille, 1809 (12 species)

1. ⸗ Chelostoma campanularum Kirby, 1802 in Stanisavljević (2000); AĐ coll. [LC]
2. Chelostoma distinctum Stoeckhert, 1929 in Kuhlmann et al. (2020); Stanisavljević (2000); as Eriades distinctus Stoeckhert, 1929 in Živojinović (1950). [LC]
3. ⸗ Chelostoma emarginatum Nylander, 1856 in Kuhlmann et al. (2020); AĐ coll.; also as Chelostoma appendiculatum (Morawitz, 1871) in Stanisavljević (2000); as Eriades emarginatus Nylander, 1856 and also as Eriades appendiculatus in Vorgin (1918). [LC]
4. ⸗ Chelostoma florisomne Linnaeus, 1758 in Kuhlmann et al. (2020); Stanisavljević (2000); AĐ coll.; SG coll.; as Chelostoma florisomnis in Mocsáry (1897); as Eriades maxillosus (Linnaeus, 1758) in Rafajlović and Seleši (1958); Živojinović (1950); as Eriades florisomnis Spinola in Vorgin (1918); Apfelbeck (1896). [LC]
5. ▪ Chelostoma foveolatum Morawitz, 1868 in Kuhlmann et al. (2020). [LC]
6. ▪ Chelostoma grande Nylander, 1852 in Kuhlmann et al. (2020). [DD]
7. Chelostoma handlirschi Schletterer, 1889 as Eriades handlirschi (Schletterer, 1889) in Rafajlović and Seleši (1958); Lebedev (1931). Note: According to Müller (2015) there are possible errors in older literature records regarding identification and distribution (“Reliable records exist only for Romania and Bulgaria”) of this species. [DD]
8. ▪ Chelostoma mocsaryi Schletterer, 1889 in Kuhlmann et al. (2020). [LC]
9. ⸗ Chelostoma nasutum Pérez, 1895 New material examined: 1 ♀; Vlasina Rid; 42.7253°N, 22.3284°E; 22–23 Jul. 2019; A. Đukić leg.; Jelle Devalez det.; AĐ coll. [LC]
10. ⸗ Chelostoma rapunculi Lepeletier, 1841 in Kuhlmann et al. (2020); Stanisavljević (2000); AĐ coll.; as Eriades nigricornis Nylander, 1848 in Rafajlović and Seleši (1958); Lebedev (1931). [LC]
11. ▪ Chelostoma styriacum Schwarz & Gusenleitner, 1999 in Kuhlmann et al. (2020). [LC]
12. ▪ Chelostoma ventrale Schletterer, 1889 in Kuhlmann et al. (2020). [LC]

*Coelioxys* Latreille, 1809 (17 species)

1. ▪ Coelioxys acanthura Illiger, 1806 in Kuhlmann et al. (2020). [DD]
2. ⸗ Coelioxys afer Lepeletier, 1841 as Coelioxys afra Lepeletier, 1841 in Kuhlmann et al. (2020); Stanisavljević (2000); Rafajlović and Seleši (1958); Lebedev (1931); Vorgin (1918); Mocsáry (1897); AĐ coll.; SG coll.; as Coelioxys coronata Förster, 1853 in Petrik (1958). [LC]
3. Coelioxys alatus Förster, 1853 as Coelioxys alata Förster, 1853 in Kuhlmann et al. (2020); Stanisavljević (2000); Živojinović (1950). [LC]
4. ⸗ Coelioxys argenteus Lepeletier, 1841 as Coelioxys argentea Lepeletier, 1841 in Kuhlmann et al. (2020); Stanisavljević (2000); ZM coll. [LC]
5. ⸗ Coelioxys aurolimbatus Förster, 1853 as Coelioxys aurolimbata Förster, 1853 in Kuhlmann et al. (2020); Stanisavljević (2000); Rafajlović and Seleši (1958); Lebedev (1931); Vorgin (1918); FSUNS. [LC]
6. Coelioxys brevis Eversmann, 1852 in Stanisavljević (2000); Rafajlović and Seleši (1958); as Coelioxys erythropyga Förster, 1853 in Petrik (1958); Mocsáry (1897). [LC]
7. Coelioxys conoideus (Illiger, 1806) as Coelioxys conoidea Illiger, 1806 in Kuhlmann et al. (2020); Stanisavljević (2000); Rafajlović and Seleši (1958); Živojinović (1950). [LC]
8. Coelioxys echinatus Förster, 1853 as Coelioxys echinata Förster, 1853 in Kuhlmann et al. (2020); Stanisavljević (2000); as Coelioxys rufocaudata Smith, 1854 in Rafajlović and Seleši (1958); Mocsáry (1897). [LC]
9. ⸗ Coelioxys elongatus Lepeletier, 1841 as Coelioxys elongata Lepeletier, 1841 in Kuhlmann et al. (2020); ZM coll. [LC]
10. Coelioxys emarginatus Förster, 1853 as Coelioxys emarginata Förster, 1853 in Stanisavljević (2000); Petrik (1958); Rafajlović and Seleši (1958); Mocsáry (1897). [LC]
11. Coelioxys haemorrhoa Förster, 1853 in Stanisavljević (2000); Živojinović (1950). [LC]
12. Coelioxys inermis Kirby, 1802 in Kuhlmann et al. (2020); Stanisavljević (2000); as Coelioxys acuminata Nylander, 1852 in Rafajlović and Seleši (1958); Živojinović (1950); Lebedev (1931). [LC]
13. Coelioxys mandibularis Nylander, 1848 in Kuhlmann et al. (2020); Stanisavljević (2000). [LC]
14. ⸗ Coelioxys obtusus Pérez, 1884 as Coelioxys obtusa Pérez, 1884 in Kuhlmann et al. (2020); Stanisavljević (2000); SG coll. [LC]
15. ⸗ Coelioxys polycentris Förster, 1853 in Kuhlmann et al. (2020); Stanisavljević (2000); Petrik (1958); Rafajlović and Seleši (1958); Vorgin (1918); Mocsáry (1897); SG coll. [LC]
16. ⸗ Coelioxys quadridentatus (Linnaeus, 1758) in Krunić et al. (1988); ZM coll.; as Coelioxys quadridentata (Linnaeus, 1758) in Kuhlmann et al. (2020); Stanisavljević (2000). [LC]
17. ⸗ Coelioxys rufescens Lepeletier and Audinet-Serville, 1825 in Kuhlmann et al. (2020); Stanisavljević (2000); Rafajlović and Seleši (1958); Živojinović (1950); Lebedev (1931); Vorgin (1918); Mocsáry (1897); ZM coll. [LC]

*Heriades* Spinola, 1808 (2 species)

1. ⸗ Heriades crenulata Nylander, 1856 in Kuhlmann et al. (2020); as Heriades crenulatus Nylander, 1856 in Mudri-Stojnić (2018); Mudri-Stojnić et al. (2012); Stanisavljević (2000); Krunić et al. (1988); as Eriades crenulatus in Rafajlović and Seleši (1958); Vorgin (1955); Mocsáry (1897). [LC]
2. ⸗ Heriades truncorum Linnaeus, 1758 in Stanisavljević (2000); Živojinović (1950); Lebedev (1931); AĐ coll.; FSUNS; as Eriades truncorum (Linnaeus) in Rafajlović and Seleši (1958); Mocsáry (1897). [LC]

*Hofferia* Tkalců, 1984 (1 species)

1. Hofferia schmiedeknechti Schletterer, 1889 in Stanisavljević (2000). [LC]

*Hoplitis* Klug, 1807 (17 species)

1. Hoplitis acuticornis Dufour & Perris, 1840 in Stanisavljević (2000); as Osmia acuticornis Dufour & Perris, 1840 in Rafajlović and Seleši (1958). [LC]
2. ⸗ Hoplitis adunca Panzer, 1798 in Kuhlmann et al. (2020); Markov (2017); Stanisavljević (2000); AĐ coll.; as Hoplites in Krunić et al. (1988); as Osmia adunca (Panzer, 1798) in Petrik (1958); Rafajlović and Seleši (1958); Živojinović (1950); as Osmia spinolae Lepeletier, 1841 in Mocsáry (1897). [LC]
3. Hoplitis anthocopoides Schenck, 1853 in Stanisavljević (2000); as Osmia caementaria Gerstäcker, 1869 in Rafajlović and Seleši (1958). [LC]
* Hoplitis bisulca Gerstäcker, 1869 as Osmia bisulca Gerstäcker, 1869 in Mocsáry (1897). [LC]
5. ▪ Hoplitis campanularis Morawitz, 1877 in Kuhlmann et al. (2020). [LC]
6. ⸗ Hoplitis claviventris (Thomson, 1872) New material examined: 1 ♀; Vlasina Rid; 42.7253°N, 22.3284°E; 21 Jul. 2019; A. Đukić leg.; Andrej Gogala det.; AĐ coll. [LC]
7. ⸗ Hoplitis dalmatica Morawitz, 1871 in SG coll.; as Anthocopa dalmatica (Morawitz, 1871) in Stanisavljević (2000); as Osmia dalmatica Morawitz, 1871 in Živojinović (1950). [LC]
8. ▪ Hoplitis illyrica Noskiewicz, 1926 in Kuhlmann et al. (2020). [LC]
9. ⸗ Hoplitis laevifrons Morawitz, 1872 in Kuhlmann et al. (2020); ZM coll. [LC]
10. ⸗ Hoplitis lepeletieri Pérez, 1879 in Stanisavljević (2000); SG coll. [LC]
11. ⸗ Hoplitis leucomelana Kirby, 1802 in Stanisavljević (2000); AĐ coll.; SG coll.; as Osmia parvula Dufour & Perris, 1840 in Rafajlović and Seleši (1958). [LC]
12. ⸗ Hoplitis loti Morawitz, 1867 in Stanisavljević (2000); SG coll. [LC]
13. Hoplitis manicata Morice, 1901 in Kuhlmann et al. (2020); Stanisavljević (2000); as Hoplites in Krunić et al. (1988). [LC]
14. ▪ Hoplitis perezi Ferton, 1895 in Kuhlmann et al. (2020). [LC]
15. ▪ Hoplitis praestans Morawitz, 1893 in Kuhlmann et al. (2020). [LC]
16. ⸗ Hoplitis tridentata Dufour & Perris, 1840 in Stanisavljević (2000); SG coll. [LC]
17. ⸗ Hoplitis tuberculata Nylander, 1848 in Stanisavljević (2000); SG coll. [LC]

*Icteranthidium* Michener, 1948 (2 species)

1. ▪ Icteranthidium grohmanni Spinola, 1838 in Kuhlmann et al. (2020). [LC]
2. Icteranthidium laterale Latreille, 1809 in Stanisavljević (2000); as Anthidium laterale Latreille, 1809 in Petrik (1958); Rafajlović and Seleši (1958); Mocsáry (1897). [LC]

*Lithurgus* Latreille, 1825 (2 species)

1. Lithurgus chrysurus Fonscolombe, 1834 in Kuhlmann et al. (2020); Stanisavljević (2000); Krunić et al. (1988); Rafajlović and Seleši (1958); Živojinović (1950); Lebedev (1931); Mocsáry (1897); Korlević (1890). [LC]
2. ⸗ Lithurgus cornutus Fabricius, 1787 in Kuhlmann et al. (2020); Mudri-Stojnić (2018); Mudri-Stojnić et al. (2012); ZM coll.; also as Lithurgus cornutus fuscipennis Lepeletier, 1841 in Stanisavljević (2000); as Lithurgus cornuta ssp. fuscipennis Lep. in Krunić et al. (1988); as Lithurgus fuscipennis Lepeletier, 1841 Rafajlović and Seleši (1958); Živojinović (1950); Mocsáry (1897). [LC]

*Megachile* Latreille, 1802 (34 species)

1. ⸗ Megachile albisecta Klug, 1817 in Kuhlmann et al. (2020); Mudri-Stojnić (2018); Markov (2017); Mudri-Stojnić et al. (2012); as Megachile sericans Fonscolombe, 1832 in Grozdanić (1971b); Vasić (1968); Mocsáry (1897); as Creightonella albisecta (Klug, 1817) in Stanisavljević (2000); SG coll. [LC]
2. ▪ Megachile albocristata Smith, 1853 in Kuhlmann et al. (2020). [LC]
3. ▪ Megachile alpicola Alfken, 1924 in Kuhlmann et al. (2020). [DD]
4. ⸗ Megachile apicalis Spinola, 1808 in Kuhlmann et al. (2020); Mudri-Stojnić (2018); Markov (2017); Markov et al. (2016); Mudri-Stojnić et al. (2012); Stanisavljević (2000); Rafajlović and Seleši (1958); Lebedev (1931); Vorgin (1918); Mocsáry (1897). [LC]
5. Megachile bombycina Radoszkowski, 1874 in Stanisavljević (2000); Rafajlović and Seleši (1958); Vorgin (1955); Živojinović (1950); Mocsáry (1897). [DD]
6. ▪ Megachile burdigalensis Benoist, 1940 in Kuhlmann et al. (2020). [DD]
7. ⸗ Megachile centuncularis Linnaeus, 1758 in Kuhlmann et al. (2020); Markov (2017); Markov et al. (2016); Stanisavljević (2000); Krunić et al. (1988); Grozdanić and Vasić (1966a); Rafajlović and Seleši (1958); Živojinović (1950); Lebedev (1931); Mocsáry (1897); AĐ coll.; ZM coll. [LC]
8. Megachile circumcincta Kirby, 1802 in Kuhlmann et al. (2020); Stanisavljević (2000); Rafajlović and Seleši (1958). [LC]
9. ⸗ Megachile concinna Smith, 1879 in Kuhlmann et al. (2020); as Megachile argentata (Fabricius, 1793) in Stanisavljević (2000); Petrik (1958); Rafajlović and Seleši (1958); Živojinović (1950); Lebedev (1931); Mocsáry (1897); ZM Coll. [DD]
10. ⸗ Megachile deceptoria Pérez 1890 in Kuhlmann et al. (2020); FSUNS. [DD]
11. ⸗ Megachile ericetorum Lepeletier, 1841 in Kuhlmann et al. (2020); Mudri-Stojnić (2018); Markov (2017); Markov et al. (2016); Grozdanić (1971b); Grozdanić and Mučalica (1968a); Grozdanić and Vasić (1966a); Rafajlović and Seleši (1958); Vorgin (1918); as Chalicodoma ericetorum (Lepeletier, 1841) in Stanisavljević (2000); Krunić et al. (1988); ZM coll. [LC]
12. Megachile flabellipes Pérez, 1895 in Kuhlmann et al. (2020); Stanisavljević (2000). [DD]
13. Megachile fulvimana Eversman, 1852 in Stanisavljević (2000). [DD]
14. ⸗ Megachile genalis Morawitz, 1880 in Kuhlmann et al. (2020); Stanisavljević (2000); ZM coll. [DD]
15. ▪ Megachile giraudi Gerstäcker, 1869 in Kuhlmann et al. (2020). [DD]
16. Megachile hungarica Mocsáry, 1877 as Chalicodoma hungarica Mocsáry, 1877 in Stanisavljević (2000). [DD]
17. Megachile lagopoda Linnaeus, 1761 in Kuhlmann et al. (2020); Stanisavljević (2000); Rafajlović and Seleši (1958); Živojinović (1950); Lebedev (1931); Mocsáry (1897). [LC]
18. ⸗ Megachile leachella Curtis, 1828 in Kuhlmann et al. (2020); Markov (2017); Markov et al. (2016); ZM coll. [LC]
19. Megachile leucomalla Gerstäcker, 1869 in Stanisavljević (2000); Petrik (1958); Mocsáry (1897). [DD]
20. Megachile ligniseca Kirby, 1802 in Kuhlmann et al. (2020); Stanisavljević (2000); Krunić et al. (1988); Rafajlović and Seleši (1958); Živojinović (1950); Lebedev (1931); Vorgin (1918). [DD]
21. ▪ Megachile manicata Giraud, 1861 in Kuhlmann et al. (2020). [DD]
22. ⸗ Megachile maritima Kirby, 1802 in Kuhlmann et al. (2020); Markov (2017); Markov et al. (2016); Stanisavljević (2000); Krunić et al. (1988); Petrik (1958); Rafajlović and Seleši (1958); Lebedev (1931); Mocsáry (1897). [DD]
23. ⸗ Megachile melanopyga Costa, 1863 in Kuhlmann et al. (2020); Mudri-Stojnić (2018); Stanisavljević (2000); Rafajlović and Seleši (1958). [LC]
24. ▪ Megachile nigriventris Schenck, 1870 in Kuhlmann et al. (2020). [DD]
25. Megachile octosignata Nylander, 1852 in Kuhlmann et al. (2020); Lebedev (1931). [DD]
26. Megachile parietina Geoffroy, 1785 in Kuhlmann et al. (2020); also as Chalicodoma muraria auct. in Apfelbeck (1896); as Chalicodoma parietina (Geoffroy, 1785) in Stanisavljević (2000); Krunić et al. (1988). [LC]
27. ⸗ Megachile pilicrus Morawitz, 1877 in Kuhlmann et al. (2020); Markov (2017); Markov et al. (2016); Stanisavljević (2000); Rafajlović and Seleši (1958); Živojinović (1950); AZ coll. [DD]
28. ⸗ Megachile pilidens Alfken, 1924 in Kuhlmann et al. (2020); Mudri-Stojnić (2018); Markov (2017); Markov et al. (2016); Stanisavljević (2000); Grozdanić (1971b); Lebedev (1931); ZM coll. [LC]
29. ▪ Megachile pyrenaea Pérez, 1890 in Kuhlmann et al. (2020). [DD]
30. ⸗ Megachile pyrenaica Lepeletier, 1841 in Kuhlmann et al. (2020); as Chalicodoma pyrenaica (Lepeletier, 1841) in Stanisavljević (2000); ZM coll. [DD]
31. ⸗ Megachile rotundata Fabricius, 1793 in Kuhlmann et al. (2020); Stanisavljević and Tomanović (2006); Stanisavljević (2000); Krunić et al. (1997, 1995b, 1992b, 1992c, 1985, 1988); Mihajlović et al. (1989); Rafajlović and Seleši (1958); ZM coll.; as Megachile pacifica (Panzer, 1798) in Vorgin (1918); Mocsáry (1897). [DD]
32. ⸗ Megachile sculpturalis Smith, 1853 in Ćetković and Plećaš (2017). New material examined: 1 ♂, 1 ♀; Bački Maglić; 45.3686°N, 19.5381°E; 20 Jul. 2019; B. Šikoparija leg.; Sonja Mudri-Stojnić det.; FSUNS.
33. ⸗ Megachile versicolor Smith, 1844 in Kuhlmann et al. (2020); Stanisavljević (2000); Rafajlović and Seleši (1958); AĐ coll. [DD]
34. ⸗ Megachile willughbiella Kirby, 1802 in Kuhlmann et al. (2020); Markov (2017); Markov et al. (2016); Mučalica and Stanisavljević (2005); Stanisavljević (2000); Petrik (1958); Rafajlović and Seleši (1958); Živojinović (1950); Lebedev (1931); Mocsáry (1897); AĐ coll.; ZM coll. [LC]

*Osmia* Panzer, 1806 (26 species)

1. ⸗ Osmia andrenoides Spinola, 1808 in Kuhlmann et al. (2020); Rafajlović and Seleši (1958); Mocsáry (1897); Apfelbeck (1896); as Anthocopa andrenoides Spinola, 1808 in Stanisavljević (2000); Krunić et al. (1988); SG coll. [LC]
2. ▪ Osmia apicata Smith, 1853 in Kuhlmann et al. (2020). [LC]
3. ⸗ Osmia aurulenta Panzer, 1799 in Kuhlmann et al. (2020); Mudri-Stojnić (2018); Markov (2017); Markov et al. (2016); Mudri-Stojnić et al. (2012); Stanisavljević (2000); Krunić et al. (1988); Grozdanić (1971b); Grozdanić and Vasić (1965c); Rafajlović and Seleši (1958); Vorgin (1955); Lebedev (1931); SG coll. [LC]
4. ⸗ Osmia bicolor Schrank, 1781 in Kuhlmann et al. (2020); Markov (2017); Markov et al. (2016); Stanisavljević (2000); Grozdanić (1971b, 1965); Grozdanić and Vasić (1965a, 1965c); Rafajlović and Seleši (1958); Lebedev (1931); SG coll. [LC]
5. ⸗ Osmia bicornis Linnaeus, 1758 in Markov (2017); Markov et al. (2016); Grozdanić and Vasić (1966a); Vorgin (1955); Grozdanić (1928); AĐ coll.; as Osmia rufa (Linnaeus, 1758) in Krunić and Stanisavljević (2006a, 2006b, 2006c, 2006d; 2000; 1996); Krunić et al. (2005, 1999, 1998, 1996, 1995a); Stanisavljević et al. (1999, 1997a, 1997b); Kulinčević et al. (1997); Grozdanić (1971b, 1960); Rafajlović and Seleši (1958); Živojinović (1950); Lebedev (1931); ZM coll.; SG coll. [LC]
6. ⸗ Osmia bidentata Morawitz, 1876 in Kuhlmann et al. (2020); Mudri-Stojnić (2018); Müller (2018); Markov (2017); Markov et al. (2016); Grozdanić and Radivojević (1972); Grozdanić (1971b); Lebedev (1931); Mocsáry (1897); as Anthocopa bidentata (Morawitz, 1876) in Stanisavljević (2000); Krunić et al. (1988); as Osmia affinis Frivaldszky, 1877 in Petrik (1958); as Hoplosmia bidentata (Morawitz, 1876) in SG coll. [LC]
7. ⸗ Osmia brevicornis Fabricius, 1798 in Kuhlmann et al. (2020); Markov (2017); Markov et al. (2016); Stanisavljević (2000); as Osmia atrocaerulea Schilling, 1849 in Rafajlović and Seleši (1958); as Osmia panzeri Morawitz, 1869 in Mocsáry (1897). [LC]
8. ⸗ Osmia caerulescens Linnaeus, 1758 in Kuhlmann et al. (2020); Markov (2017); Markov et al. (2016); Stanisavljević (2000); Krunić et al. (1988); Grozdanić and Vasić (1966a); Rafajlović and Seleši (1958); AĐ coll.; SG coll.; as Osmia aenea Linnaeus, 1761 in Živojinović (1950); Lebedev (1931). [LC]
9. Osmia cephalotes Morawitz, 1870 as Osmia cephalotes longiceps Morawitz, 1876 in Stanisavljević (2000). [LC]
10. ⸗ Osmia cornuta Latreille, 1805 in Markov (2017); Markov et al. (2016); Stanisavljević et al. (2013, 2000a, b, 1999, 1997a, b); Stanisavljević (2009, 2000, 1996); Krunić and Stanisavljević (2006a, b, , d); Maccagnani et al. (2007); Krunić et al. (2005, 2001, 1999, 1998, 1996, 1995a, 1992a, 1991, 1989, 1988); Kulinčević et al. (1997); Rafajlović and Seleši (1958); Lebedev (1931); AĐ coll.; AZ coll.; ZM coll. [LC]
11. ▪ Osmia croatica Friese, 1893 in Kuhlmann et al. (2020). [LC]
12. ⸗ Osmia emarginata Lepeletier, 1841 in Stanisavljević (2000); ZM coll. [LC]
13. ▪ Osmia erythrogastra Ferton, 1905 in Kuhlmann et al. (2020). [LC]
14. Osmia gallarum Spinola, 1808 in Stanisavljević (2000); Lebedev (1931). [LC]
15. ▪ Osmia jason Benoist, 1929 in Kuhlmann et al. (2020). [LC]
16. ⸗ Osmia leaiana Kirby, 1802 in Stanisavljević (2000); Rafajlović and Seleši (1958); Živojinović (1950); Lebedev (1931); AĐ coll.; as Osmia solskyi Morawitz, 1870 in Mocsáry (1897). [LC]
17. ⸗ Osmia melanogaster Spinola, 1808 in Kuhlmann et al. (2020); Markov (2017); Markov et al. (2016); Stanisavljević (2000); as Osmia melanogastra in Rafajlović and Seleši (1958); Mocsáry (1897); as Osmia aterrima Morawitz, 1872 in Lebedev (1931). [LC]
18. ⸗ Osmia niveata Fabricius, 1804 in Kuhlmann et al. (2020); Markov (2017); Markov et al. (2016); as Osmia fulviventris (Panzer, 1798) in Stanisavljević (2000); Krunić et al. (1988); Rafajlović and Seleši (1958); Vorgin (1955); Živojinović (1950); Lebedev (1931); Apfelbeck (1896). [LC]
19. ▪ Osmia padri Tkalcu, 1974 in Kuhlmann et al. (2020). [DD]
20. ▪ Osmia pilicornis Smith, 1846 in Kuhlmann et al. (2020). [LC]
21. ⸗ Osmia rufohirta Latreille, 1811 in Kuhlmann et al. (2020); Markov (2017); Markov et al. (2016); Grozdanić (1971b, 1969b); Grozdanić and Vasić (1965c); Rafajlović and Seleši (1958); Živojinović (1950); Mocsáry (1897); SG coll; as Hoplitis rufohirta (Latreille, 1811) in Stanisavljević (2000); as Hoplites rufohirta (Latr.) in Krunić et al. (1988). [LC]
22. ▪ Osmia saxicola Ducke, 1899 in Kuhlmann et al. (2020). [LC]
23. ▪ Osmia signata Erichson, 1835 in Kuhlmann et al. (2020). [LC]
24. ⸗ Osmia spinulosa Kirby, 1802 in Mudri-Stojnić (2018); Mudri-Stojnić et al. (2012); Rafajlović and Seleši (1958); Vorgin (1955); as Anhocopa spinulosa (Kirby, 1802) in Stanisavljević (2000). [LC]
25. Osmia submicans Morawitz, 1870 in Stanisavljević (2000); Rafajlović and Seleši (1958). [LC]
26. Osmia versicolor Latreille, 1811 in Stanisavljević (2000); Apfelbeck (1896). [LC]

*Protosmia* Ducke, 1900 (1 species)

1. Protosmia longiceps Friese, 1899 as Eriades longiceps Friese, 1899 in Vorgin (1918). [DD]

*Pseudoanthidium* Friese, 1898 (3 species)

1. Pseudoanthidium scapulare (Latreille, 1809) as Paranthidiellum lituratum (Panzer, 1801) in Stanisavljević (2000); Krunić et al. (1988); as Anthidium lituratum (Panzer, 1801) in Rafajlović and Seleši (1958); Vorgin (1918). [DD]
* Pseudoanthidium reticulatum Mocsáry, 1884 as Anthidium mocsaryi Friese, 1897 in Mocsáry (1897). [DD]
* Pseudoanthidium tenellum Mocsáry, 1881 as Anthidium tenellum Mocsáry, 1881 in Mocsáry (1897). [DD]

*Rhodanthidium* Isensee, 1927 (2 species)

1. Rhodanthidium septemdentatum Latreile, 1809 in Kuhlmann et al. (2020); Stanisavljević (2000); as Anthidium septemdentatum Latreille, 1809 in Grozdanić and Vasić (1965c); Rafajlović and Seleši (1958); Živojinović (1950); Lebedev (1931). [DD]
2. ▪ Rhodanthidium sticticum Fabricius, 1787 in Kuhlmann et al. (2020). [DD]

*Stelis* Panzer, 1806 (10 species)

1. Stelis annulata Lepeletier, 1841 in Stanisavljević (2000); as Stelis frey-gessneri Friese, 1885 in Rafajlović and Seleši (1958). [DD]
2. Stelis breviuscula Nylander, 1848 in Kuhlmann et al. (2020); Stanisavljević (2000); Rafajlović and Seleši (1958); Lebedev (1931); Vorgin (1918). [LC]
3. Stelis minuta Lepeletier and Audinet-Serville, 1825 in Kuhlmann et al. (2020); Stanisavljević (2000); Rafajlović and Seleši (1958). [LC]
4. ▪ Stelis nasuta Latreille, 1809 in Kuhlmann et al. (2020). [LC]
5. ▪ Stelis odontopyga Noskiewicz, 1926 in Kuhlmann et al. (2020). [LC]
6. Stelis ornatula Klug, 1807 in Kuhlmann et al. (2020); Apfelbeck (1896). [LC]
7. Stelis phaeoptera Kirby, 1802 in Kuhlmann et al. (2020); Stanisavljević (2000); Rafajlović and Seleši (1958); Živojinović (1950); Lebedev (1931). [DD]
8. Stelis punctulatissima Kirby, 1802 in Kuhlmann et al. (2020); Stanisavljević (2000); as Stelis aterrima (Panzer, 1798) in Rafajlović and Seleši (1958); Lebedev (1931); Mocsáry (1897). [LC]
9. ⸗ Stelis signata Latreille, 1809 in Kuhlmann et al. (2020); Markov (2017); Markov et al. (2016); Stanisavljević (2000); Rafajlović and Seleši (1958). [LC]
10. Stelis simillima Morawitz, 1876 in Kuhlmann et al. (2020); Kasparek (2015). [LC]

*Trachusa* Panzer, 1804 (5 species)

1. ⸗ Trachusa byssina Panzer, 1798 in Kuhlmann et al. (2020); Stanisavljević (2000); Lebedev (1931); ZM coll. [LC]
2. ▪ Trachusa dumerlei Warncke, 1980 in Kuhlmann et al. (2020). [LC]
3. Trachusa interrupta Fabricius, 1781 in Kuhlmann et al. (2020); Kasparek (2017); as Paraanthidium interruptum (Fabricius, 1781) in Stanisavljević (2000); as Anthidium interruptum (Fabricius, 1781) in Rafajlović and Seleši (1958); Mocsáry (1897). [EN]
4. ▪ Trachusa laticeps Morawitz, 1873 in Kuhlmann et al. (2020). [NT]
5. Trachusa pubescens Morawitz, 1872 in Kasparek (2017); as Archianthidium pubescens Morawitz, 1872 in Stanisavljević (2000); as Anthidium pubescens Morawitz, 1872 in Petrik (1958); Rafajlović and Seleši (1958); Živojinović (1950); Mocsáry (1897). [DD] Note: According to Kasparek (2018), Trachusa pubescens sensu lato is a complex of five species, and specimens from Serbia belong to the newly described Trachusa balcanica Kasparek, 2018.

### Mellitidae (3 genera; 13 species)

*Dasypoda* Latreille, 1802 (4 species)

1. Dasypoda argentata Panzer, 1809 in Rafajlović and Seleši (1958); Mocsáry (1897). [NT]
2. ▪ Dasypoda braccata Eversmann, 1852 in Kuhlmann et al. (2020). [EN]
3. ⸗ Dasypoda hirtipes (Fabricius, 1793) in Kuhlmann et al. (2020); Mudri-Stojnić (2018); Markov (2017); Markov et al. (2016); AĐ coll.; also as Dasypoda plumipes Panzer, 1797 in Petrik (1958); as Dasypoda plumipes in Grozdanić (1971b); Rafajlović and Seleši (1958); Vorgin (1955, 1918); Živojinović (1950); Lebedev (1931); Mocsáry (1897); ZM coll. [LC]
4. ⸗ Dasypoda pyrotrichia Förster, 1855 New material examined: 2 ♀♀; Vlasina, Dejanova reka; 42.6888°N, 22.3954°E; 24 Jul. 2019; A. Đukić leg.; Denis Michez det.; AĐ coll. [LC]

*Macropis* Panzer, 1809 (3 species)

1. ⸗ Macropis europaea Warncke, 1973 in Kuhlmann et al. (2020); AĐ coll.; as Macropis labiata (Fabricius, 1804) in Apfelbeck (1896). [LC]
2. Macropis frivaldszkyi Mocsáry, 1878 in Kuhlmann et al. (2020); Vorgin (1918); Mocsáry (1897). [NT]
3. Macropis fulvipes (Fabricius, 1804) in Kuhlmann et al. (2020); Lebedev (1931). [LC]

*Melitta* Kirby, 1802 (6 species)

1. Melitta budensis (Mocsary, 1878) in Grozdanić (1971b); Rafajlović and Seleši (1958). [LC]
* Melitta dimidiata Morawitz, 1876 in Apfelbeck (1896). [NT]
3. ⸗ Melitta haemorrhoidalis (Fabricius, 1775) in Kuhlmann et al. (2020); Mudri-Stojnić (2018); Rafajlović and Seleši (1958); Živojinović (1950). [LC]
4. ⸗ Melitta leporina (Panzer, 1799) in Kuhlmann et al. (2020); Mudri-Stojnić (2018); Rafajlović and Seleši (1958); Živojinović (1950); Morawitz (1876); Apfelbeck (1896). [LC]
5. ⸗ Melitta nigricans Alfken, 1905 in Kuhlmann et al. (2020); Rafajlović and Seleši (1958); Živojinović (1950); AĐ coll.; AZ coll. [LC]
6. Melitta tricincta Kirby, 1802 in Kuhlmann et al. (2020); Rafajlović and Seleši (1958). [NT]

## Discussion

Of the 706 species from six families of bees presented here for Serbia, more than half (53%) belong to only two families of the group of long-tongued bees, i.e. Apidae (32%) and Megachilidae (21%). Apidae is also the family represented with most genera, 31% of the total number of 58. The genus most rich in species is *Andrena* (fam. Andrenidae) with 104 reported species, followed by *Nomada* (fam. Apidae) – 77 and *Lasioglossum* (fam. Halictidae) – 72 species. Among all genera, 26 (44.8%) are represented with only one or two species. Regarding families, the lowest number of species is recorded within Mellitidae, only 13 (1.8%).

According to the first Red List of European bees (Nieto et al. 2014) and its addition (Rasmont et al. 2017), the pattern found here is similar to that at the European level (Table 1). The first checklist included 1,965 native European bee species, whereas the update presented 2,051 species for Europe and gave the first estimation of 3,408 species for the West Palaearctic biogeographical region. The most prominent and diverse bee family in Europe / West Palaearctic is the Apidae (28.1% / 27.2% of species), while the least diverse is Mellitidae (with only 1.9% / 1.7%). Considering there are approximately 20,000 bee species worldwide, Serbia hosts 3.5% of the total, 20.7% of Western-Palaearctic, and 34.4% of the European bee diversity, according to the list we present in this study. Regarding bee genera, more than half of Western-Palaearctic, and the majority of the European ones are represented in our list, 58 out of 105 and 77, respectively, the latter number updated form 75 (Nieto et al. 2014), since *Halictus* subgenera *Seladonia* and *Vestitohalictus* have recently been erected as distinct genera (Rasmont et al. 2017 and references therein).

For most of the species listed here, newer records (starting with year 2000) have been found in various bibliographic sources and collections. However, for 97 species there are no publicly available records from the 21^st^ century. Furthermore, for 15 of these species the only found data of presence in Serbia are from the 19^th^ century, most of them reported only once, therefore the current presence of these species in the given localities is not certain. Our knowledge of bee fauna is still somewhat fragmentary and uneven among different parts of Serbia, since many localities remain understudied or were investigated a long time ago. A comprehensive future research is needed in order to confirm and update the data in this provisional list that is meant to represent a review of so far published records and a basis for further studies. Moreover, among 706 species, the presence in Serbia for 314 species was confirmed by determination and review of materials, while data are from literature for 392 species. A third of all the species (227) are included in our list according to only one literature source each, and for almost a quarter (153) of all the species, the only source for their occurrence in Serbia is the “Checklist of the Western Palaearctic Bees” (Kuhlmann et al. 2020). Of the 706 species recorded in total, 510 are also listed in Kuhlmann et al. (2020) as being present in Serbia. Therefore, we present 196 bee species as potential additions to the distribution maps of that checklist. Furthermore, 14 of these species are presented here as the first published records for Serbia.

According to the European Red List (Nieto et al. 2014), most species (more than half) recorded for Serbia and listed here are in the Least Concern category (55.4%), followed by those classified as Data Deficient (31.4%) since there was not enough scientific information to evaluate their risk of extinction. A further 9.1% of species have been assessed as Near Threatened. Therefore, ca. 4% of bee species present in Serbia are considered threatened in Europe; i.e., ten Vulnerable and 18 Endangered species (Table 2). Only one species, *Bombuscullumanus* (fam. Apidae), is listed as Critically endangered at the European level. Three of the species categorised as Endangered according to the European Red List have not been recorded in Serbia in the 21^st^ century. Therefore, Serbia hosts species of conservation concern in Europe; however, the current presence of some species requires re-confirmation and possible re-evaluation of their conservation status. Threatened species mostly belong to families Apidae (13: 6 VU, 6 EN, 1 CR), Colletidae (8: 3 VU, 5 EN) and Halictidae (5: 1 VU, 4 EN); there is one EN species in each of the remaining three families. The pattern is similar to that at the European level, with most threatened species in Apidae, followed by Colletidae and Halictidae. The overall proportion of threatened (VU, EN, and CR) bee species is the same (4%), but the proportional representation of Data Deficient species is higher at 56.7% (Nieto et al. 2014). Among species presented in our list, 77 not included in Kuhlmann et al. (2020) were assessed as Data Deficient (Nieto et al. 2014). Therefore, this study is an addition to the knowledge of the distribution of these species in Europe and thus a contribution to scientific information needed for their threat evaluation. Furthermore, since there is still no national Red List of bees in Serbia, the list presented here provides a baseline for future work in that direction. Only one species of the superfamily Apoidea in Serbia has been listed as protected by national law, *Bombusconfusus* (Appendix 2 of the Code on Declaration and Protection of Strictly Protected and Protected Wild Species of Plants, Animals and Fungi, Official Gazette of RS No. 5/2010, 47/2011, 32/2016 and 98/2016); species assessed as Vulnerable in Europe. Comparing global and regional Red Lists has shown that species common within their overall geographical range can be threatened on a local scale, which also highlights the importance to implement conservation measures at various geographical scales (Drossart and Gérard 2020).

Two of the species presented in our list are without the category of threat, since they are not in the European Red List (Nieto et al. 2014) but they have been included in its addition (Rasmont et al. 2017). One is *Andrenaconfinis*, previously considered a synonym of *Andrenacongruens* but now regarded as a distinct species (Rasmont et al. 2017; Kuhlmann et al. 2020). The second is *Megachilesculpturalis*, native to Eastern Asia. Until recently, European bee fauna has been without allochthonous species; however, this solitary bee has been imported, established, and is expanding rapidly, and the first record for south-east Europe was reported in Serbia in 2017 (Ćetković and Plećaš 2017).

An up-to-date species list is the foundation of biodiversity and conservation work, and knowing which species make up the diverse ecosystems will be critically important in order to protect and restore them. Bees represent one of the key components of global biodiversity, providing vital ecosystem services, being the primary pollinators of most agricultural crops and wild plants. Climate change, land-use change and other anthropogenic pressures have been affecting the diversity of bees throughout the world (Tscharntke et al. 2005; Winfree et al. 2009; Holzschuh et al. 2010, 2016; Potts et al. 2010; Gill et al. 2012; González-Varo et al. 2013; Senapathi et al. 2015). During the last decade, both scientific and public interest in the conservation of pollinators has increased considerably (Drossart and Gérard 2020). Identifying effective conservation practices for bees requires a continuous monitoring to assess their population trends and the most significant threats. The first step towards this aim is the comprehensive knowledge of bee diversity, thus the current study provides a baseline for further research in Serbia. However, our intention is to produce updates, and we hope other researchers will contribute and improve upon this list by providing new information.
